# SIRPα blockade therapy potentiates immunotherapy by inhibiting PD-L1^+^ myeloid cells in hepatocellular carcinoma

**DOI:** 10.1038/s41419-025-07779-7

**Published:** 2025-06-16

**Authors:** Da Huang, Min Xu, Hui Wang, Yufei Zhao, Zihao Zhang, Mincheng Yu, Mingqin Zhou, Jingying Pan, Hong Zeng, Zichuan Yu, Qiang Yu, Mengyuan Wu, Wenxin Xu, Binghai Zhou, Bo Zhang, Hui Li, Lei Guo, Peiyi Xie

**Affiliations:** 1 Department of Thyroid Surgery; The Second Affiliated Hospital, Jiangxi Medical College, Nanchang University, Nanchang, Jiangxi China; 2 Jiangxi Province Key Laboratory of Immunology and Inflammation, The Second Affiliated Hospital, Jiangxi Medical College, Nanchang University, Nanchang, Jiangxi China; 3 Department of Liver Surgery & Transplantation, Liver Cancer Institute, Zhongshan Hospital, Fudan University, Key Laboratory of Carcinogenesis and Cancer Invasion, Ministry of Education, Shanghai, 200032 P.R. China; 4 Department of Molecular Biology, Shanghai Sixth People’s Hospital Affiliated to Shanghai Jiao Tong University School of Medicine, Shanghai, 200032 P.R. China; 5 Department of General Surgery, Zhongshan Hospital, Fudan University, Shanghai, 200032 P.R. China; 6 Second College of Clinical Medicine, Nanchang University, Nanchang, 330000 P.R. China; 7 Department of Thoracic Surgery, Zhongshan Hospital, Fudan University, Shanghai, 200032 P.R. China; 8 Division of Hepato-Biliary-Pancreatic Surgery, Department of General Surgery, The Second Affiliated Hospital, Jiangxi Medical College, Nanchang University, Nanchang, Jiangxi China

**Keywords:** Cancer immunotherapy, Immunosuppression

## Abstract

Tumor-infiltrating myeloid cells (TIMs) are pivotal cell populations involved in the immunosuppressive tumor immune microenvironment (TIME). However, there has been little success in large-scale clinical trials of myeloid cell modulators. We aim to investigate potential molecular targets for TIMs and disclose the underlying mechanism. Using mass cytometry by time of flight (CyTOF), we analyzed 24 spontaneous HCC tissues from mouse. Orthotopic and subcutaneous tumor models were established with or without anti-SIRPα antibody treatment. Patient-derived tumor xenografts model (PDX) was used to identify the CD47-SIRPα axis blocked therapy. In 24 murine spontaneous HCC tissues, we observed that the proportion of myeloid-derived suppressor cells (MDSCs) plus macrophages accounts for 40–90% of TIMs and SIRPα was highly expressed in TIMs, especially in macrophages and MDSCs. Through in vivo experiments, we showed that anti-SIRPα therapy inhibited tumor growth, accompanied by increased CD8^+^ T cells infiltration and decreased TIMs including MDSCs and macrophages. We found that anti-SIRPα inhibited immunosuppressive function, migration and PD-L1 expression of myeloid cells. In a series of in vivo experiments, we demonstrated the anti-tumor and immune-active effect of SIRPα-blocked therapy. Mechanistically, anti-SIRPα inhibited the immunosuppressive function and PD-L1 expression of TIMs through downregulating PI3K/AKT signaling in myeloid cells. At last, anti-SIRPα enhanced the antitumor effect of anti-PD-L1 therapy in orthotopic and spontaneous murine models. Together, SIRPα blocked therapy reversed the immunosuppressive TIME, which provides a promising therapeutic rationale for increasing the efficacy of anti-PD-L1 therapy in treating HCC.

## Introduction

Hepatocellular carcinoma (HCC) is the sixth most common malignancy and the third leading cause of cancer-related death worldwide [1]. To date, molecular therapies combined with immune checkpoint inhibitors (ICIs) have significantly improved the prognosis of patients with HCC [2]. However, the combination therapeutic strategies are limited, and more than half of the HCC patients cannot benefit from these therapies [2, 3], highlighting the urgent need to identify novel combination regimens.

Tumor-infiltrating myeloid cells (TIMs) are key components in the tumor immune microenvironment (TIME), which orchestrate immunosuppressive microenvironments within tumors [4, 5]. TIMs mainly comprise myeloid-derived suppressor cells (MDSCs) and tumor-associated macrophages (TAMs), which can diversify into a spectrum of states in the tumor microenvironment (TME) [6, 7]. Within the TME, these TIMs undergo a polarization process that contributes to an anti-inflammatory state, characterized by the presence of G-MDSC and M2-like TAMs [8]. This immunosuppressive TIME impairs the antitumor immunity and has been associated with tolerance of immunotherapy [8]. Preclinical efficacy studies showed that myeloid inhibitors can ameliorate the immunosuppressive TME and promote immunotherapy efficacy [9, 10]. To date, only a few distinct strategies have been used to target immunosuppressive myeloid cells clinically, and ongoing clinical trials targeting tumor myeloid cell subsets is promising [5]. More potential myeloid cell-targeted therapies are supposed to be investigated.

Signal regulatory protein alpha (SIRPα) is a transmembrane protein expressed primarily in myeloid cells [11, 12]. It can be activated by the cluster of differentiation 47 (CD47) protein, a “don’t eat me” signal, which plays an important role in cell clearance and tissue homeostasis [12, 13]. However, from initial clinical trials, it showed that monotherapy with CD47-SIRPα axis blockade has a limited therapeutic effect at the maximum tolerated dose [14]. Despite the CD47-SIRPα axis being widely studied in cancer, its pro-tumor mechanism and blockade therapy are seldomly reported in HCC. The potential mechanism whereby CD47-SIRPα axis blockade therapy synergizes with immunotherapy in HCC needs further investigation.

Here, we aim to investigate the antitumor efficacy targeting SIRPα in TIMs, disclose the underlying mechanism and highlight a promising strategy to enhance sensitivity to immunotherapy using anti-SIRPα treatment.

## Materials and methods

### Patients and specimens

Human HCC tissues and liver tissues were obtained from pathologist-certified HCC patients who underwent curative resection at Zhongshan Hospital, Fudan University, from April 2022 to January 2023. These patients did not receive anticancer therapy before surgery, and patients with concurrent autoimmune disease, HIV or syphilis were not included.

Written informed consent and tissues from all of the patients were obtained under approval from the Ethics Committee of Zhongshan Hospital, Fudan University. The study was performed according to the Declaration of Helsinki and the International Conference on Harmonization Guidelines for Good Clinical Practice.

### Mouse models

Wild-type male C57BL/6 mice (6-week-old) and male NSG mice were purchased from the Charles River Laboratory (Beijing, China) and fed in a temperature-controlled, specific-pathogen-free (SPF) animal laboratory. All procedures using the mice were permitted by the Institutional Animal Care and Use Committee (IACUC) of Zhongshan Hospital (Shanghai, China).

For subcutaneous mouse model, 5 × 10^6^ cultured Hepa1–6 cells were subcutaneously injected into the right inguinal fold regions. Mice were sacrificed with CO_2_ at the endpoint of the experiment, and the tumors were collected and evaluated. For the orthotopic tumor model, subcutaneous tumors were resected and cut into 1-mm^3^ cubes. Mice were anesthetized using xylazine, and single tissue pieces were implanted into the liver parenchyma. Tumor volume was calculated according to the formula: 0.5 × (larger diameter) × (smaller diameter) ^2^.

Alb-Cre/Trp53^fl/fl^ mouse model of spontaneous HCC was established as previously described [15]. Transposon pT3-EF1a-Myc plasmids and sleeping beauty transposase (SB100) were obtained from Addgene (#92046) for hydrodynamic tail-vein injection (HDTVi). Trp53^fl/fl^ and Alb-cre mice (both on a C57BL/6 background) were obtained from the Jackson Laboratory (Sacramento, CA). Mice were randomly assigned to groups according to the mean tumor volume using an ultrasound imaging platform.

To construct spontaneous HCC model, wild-type male C57BL/6 mice were administered with a single dose of N-nitrosodiethylamine (DEN) (Sigma-Aldrich, St. Louis, MO) (intraperitoneal (i.p.) injection of 25 mg/kg at 15 days of age), followed by CCl_4_ (Sigma-Aldrich) treatment (starting at 8 weeks of age; 2 ml/kg, 25% dissolved in olive oil, one time per week via i.p. injection) for continuous 28 weeks as described previously described [16]. All of the tumor tissues were collected and detected by CyTOF analysis.

For the patient-derived tumor xenografts (PDX) models construction, HCC tissues from four patients were cut into pieces and implanted into the flanks of 4- to 5-week-old NSG mice under aseptic conditions. Next, 1-cm^3^ subcutaneous tumor tissues were cut into 1-mm^3^ cubes and implanted for the second time. When the P4 generation PDX model was established, the mice were injected with 1 × 10^6^ human PBMCs (huPBMCs) and T lymphocytes through the tail vein and sacrificed with CO_2_ 48 h later. CD47 antagonist TTI-621 was used to treat mice (i.p. injection; 8 mg/kg, three times a week for 3–4 weeks).

For pharmaceutical intervention, an anti-PD-L1 antibody (BE0101, BioXCell, West Lebanon, NH) (100 μg/injection/mouse), anti-SIRPα (BE0322, BioXCell), 740 Y-P (HY-P0175, MedChemExpress, New Jersey, USA, selective agonist of PI3K (100 mg/kg/day, i.p.) or GSK690693 (30 mg/kg/day) was injected intraperitoneally three times a week after tumor implantation.

### Cytometry by time of flight (CyTOF)

Mouse tumor tissues were harvested and cut into little pieces. Next, tissue pieces were dissociated using the Mouse Tumor Dissociation Kit (Miltenyi 18 Biotec #130-096-730) in C tubes according to the manufacturer’s instructions. A 70-μm strainer was used to filter the dissociated tissues, followed by centrifugation in 36% Percoll reagent (GE Healthcare, USA).

A total of 15 metal-conjugated antibodies were prepared using the Maxpar Antibody Labeling Kit (DVS Sciences, CA, USA) according to the manufacturer’s instructions (Table S1). For mass cytometry staining, 250 nM cisplatin (Fluidigm, USA) was employed to clear dead cells, and Fc receptor blocking solution was used for incubation. After incubation in surface antibodies cocktail for 30 min on ice, cells were washed twice and fixed in intercalation solution (Maxpar Fix and Perm Buffer containing 250 nM 191/193Ir, Fluidigm) overnight. The second day, an intracellular antibody cocktail was employed for staining for 30 min on ice. A Helios3 CyTOF system (Fluidigm, USA) was used to analyze the samples, and the CyTOF data were analyzed on the Cytobank platform (https://www.cytobank.org/). Data acquisition and analyses were performed by PLTTech Inc (Hangzhou, China).

### Coculture assay

A 24-mm Transwell chamber with a 0.4-µm pore polycarbonate membrane (Corning, USA) was employed to construct a coculture system between myeloid cells and HCC cells in vitro. Before coculture, Hepa1–6 or LPC-H12 cells (5 × 10^5^/well) were seeded in the lower chamber. Twenty-four hours later, the upper chamber was seeded with a total of 5 × 10^5^ isolated myeloid cells of mice or 150 nM PMA-induced THP-1 cells in RPMI 1640 medium containing 10% FBS and 100 U/L penicillin/streptomycin in a humidified incubator containing 5% CO_2_ at 37 °C. Myeloid cells with or without SIRPA overexpression were treated with anti-SIRPα mAb (5 μg/ml) or anti-CD47 mAb (10 μg/ml) compared with the control, followed by incubation with mCD47-Fc (Cat#1866-CD-050, R&D Systems) for 12 h. Cells were collected for analysis after cocultivation.

### Migration assay

The effect of the indicated HCC cells on the chemotaxis of BMDM or G-MDSCs was analyzed employing a 24-well plate containing Transwell inserts with an 8-µm pore size polyethylene membrane (Corning, USA). Myeloid cells (5 × 10^4^ /well) were placed into the upper chamber for 2 h. Then, the chambers were moved to the 24-well plates containing supernatant of the coculture system and incubated at 37 °C. After incubating for 24 h, the upper chamber was fixed in 4% paraformaldehyde and stained with 0.1% crystal violet. Next, cells on the upper surface of the chamber were gently wiped off with a cotton swab. Migrated cells on the lower surface of the chamber were imaged through the microscope. Cells in three random fields per group were counted.

### Statistical analysis

Data were analyzed through GraphPad Prism (v.9.0) (for experimental data), R (v.3.6.1), RStudio (v.3.5.3) and Python (v.3.7.4) (for sequencing data). Differences between the two groups were analyzed by Student’s *t*-test, and differences among multiple groups were evaluated using one-way ANOVA. Survival analyses were performed using log-rank tests. *P* values less than 0.05 were considered to indicate a significant difference. The results are presented as the mean ± standard deviation.

(Additional materials and methods are described in the Supplemental Materials and Methods).

## Results

### SIRPα was highly expressed in myeloid cells in HCC

**Fig. 1:**
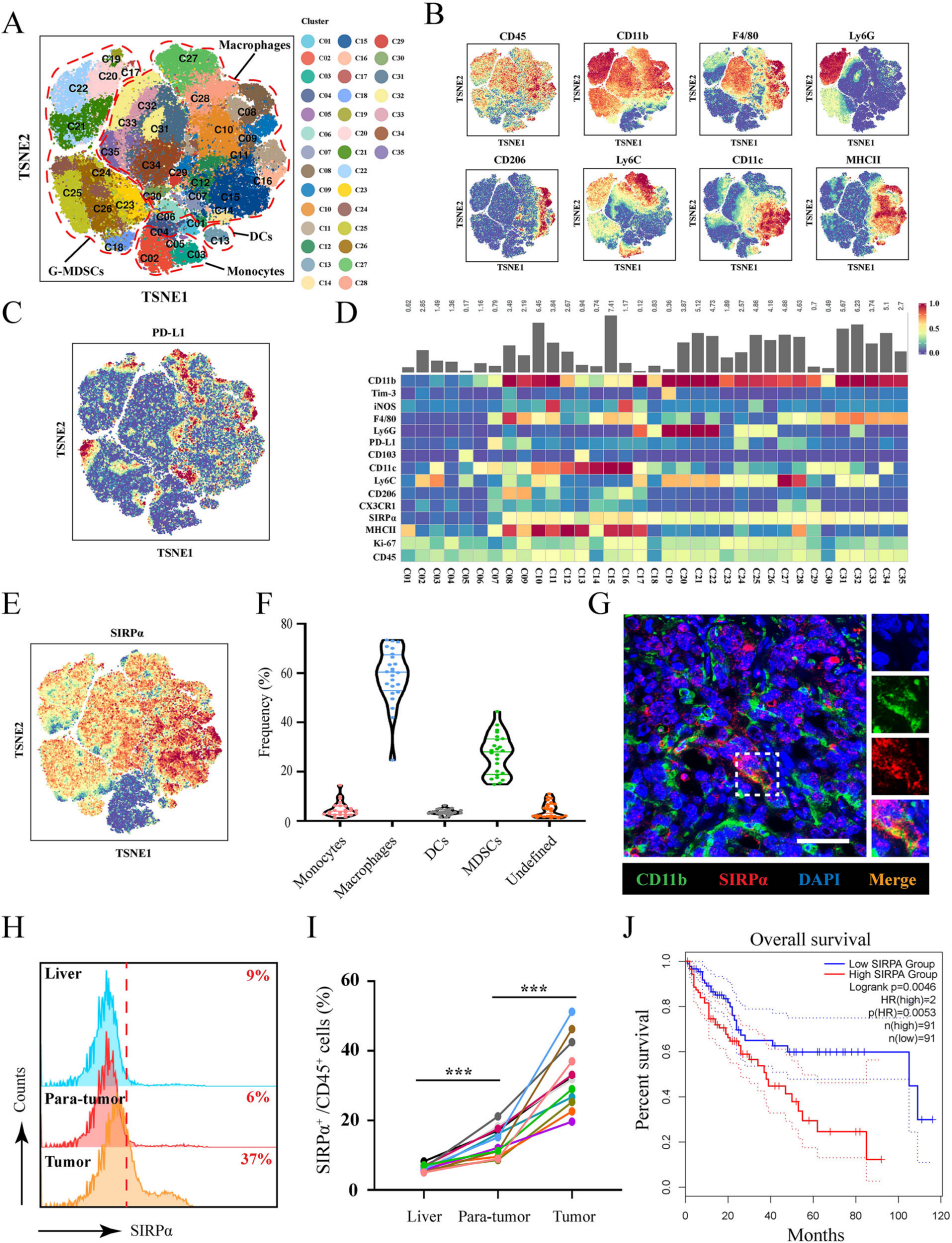
SIRPα was upregulated in TIMs and correlated with the poor prognosis of HCC patients. **A** t-SNE plot of the CyTOF data of all myeloid cells from 24 spontaneous mouse HCC tissues. **B**, **C** t-SNE plot of all myeloid cells colored according to the expression levels of CD45, CD11b, F4/80, Ly6G, CD206, Ly6C, CD11c, MHCII, and PD-L1. **D** Heatmap of 15 markers expressed in each cell cluster. **E** t-SNE plot of all myeloid cells colored according to the expression levels of SIRPα. **F** The frequency of immune cells among the CD11b^+^ cells in each group. **G** mIF analysis of human HCC tissues to detect the expression of CD11b and SIRPα. Scale bar: 100 μm. **H**, **I** Flow cytometry analysis of SIRPα expression in human liver, para-tumor and HCC tissues in CD45^+^ cells. **J** Overall survival of HCC patients in the TCGA database with high or low SIRPα expression. All data presented are shown as the mean ± SD. *P < 0.05, **P < 0.01, ***P < 0.001, ns not significant.

To determine the subset of myeloid cells (CD45^+^CD11b^+^) in HCC, we analyzed 24 DEN/CCL_4_^-^induced spontaneous murine tumor tissues using mass cytometry by time of flight (CyTOF). Through t-distributed stochastic neighbor embedding (TSNE) analysis, the expression profile analysis of 15 markers (including CD45, CD11b, F4/80, Ly6G, PD-L1, MHCII, Ki-67, CX3CR1, CD206, Ly6C, CD11c, CD103, iNOS, Tim-3, and SIRPα) identified 35 distinct cell clusters (Fig. 1A–D and Fig. S1A). The heatmap of the expression of the 15 markers in 35 clusters was shown in Fig. 1D. Our data showed that SIRPα was highly expressed in macrophages and G-MDSCs (CD11b^+^ Ly6G^+^Ly6C^low^in mouse) (Fig. 1D, E). Additionally, we found that the proportion of G-MDSCs plus macrophages accounts for 40–90% of TIMs (Fig. 1F). Furthermore, we observed that SIRPα was widely expressed in myeloid cells in human HCC tissues (Fig. 1G). Using flow cytometry, we detected HCC tissues from 12 patients, and found that SIRPα was upregulated in CD45^+^ immune cells compared with that in liver and para-tumor tissues (Fig. 1H, I). Through GEPIA2 and TIMER2.0 database [17, 18], we also found that SIRPα expression was negatively correlated with CD8^+^ T cells infiltration, and SIRPα overexpression in HCC was correlated with poor prognosis of patients (Fig. 1J and Fig. S1B, C). These findings indicated that SIRPα was highly expressed in TIMs and could be a potential molecular target in treating HCC.

### Anti-SIRPα therapy inhibited HCC progression and enhanced infiltration and function of CD8^+^ T cells in vivo

**Fig. 2:**
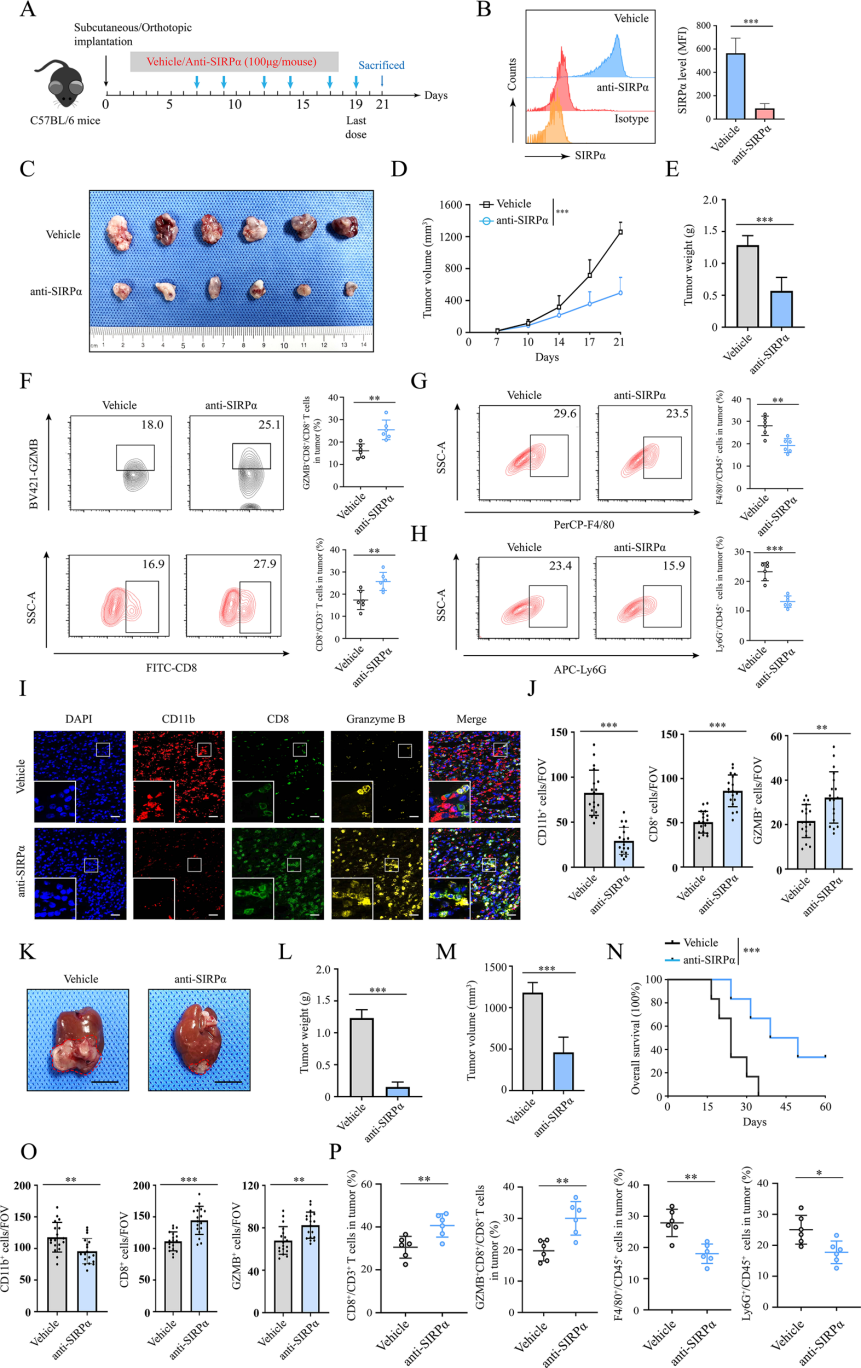
SIRPα blockade inhibited tumor growth in vivo. **A** The schematic diagram showing the establishment of the Hepa1–6 subcutaneous or orthotopic mouse model using wild-type C57BL/6 mice. Mice (*n* = 6 for each group) were treated with anti-SIRPα mAb or IgG (100 μg/mouse, i.p.). **B** At the end point of the experiments, TIMs were isolated from tumor tissues and SIRPα expression in TIMs was analyzed by flow cytometry in the indicated groups. The tumor images (**C**), tumor growth curves (**D**), and tumor weight (**E**) were analyzed. Tumor-infiltrating CD8^+^ T cells and GZMB expression (**F**), tumor-associated macrophages (F4/80) (**G**), and G-MDSCs (Ly6G) (**H**) were detected through flow cytometry. **I**, **J** IF analysis of mouse tumor tissues was performed to detect the expression of CD11b, CD8 and GZMB (Scale bar, 50 μm). Gross appearance of the liver in orthotopic tumor model (Scale bar, 1 cm) (**K**), tumor weight (**L**), tumor volume (**M**), and overall survival (**N**) of the mice were analyzed. **O** IF analysis of CD11b, CD8, and GZMB in tumor tissues. **P** Flow cytometry analysis of CD8^+^ T cells and TIMs in tumor. All data presented are shown as the mean ± SD. *P < 0.05, **P < 0.01, ***P < 0.001, ns not significant.

To investigate the function of anti-SIRPα therapy in treating HCC, we established subcutaneous and orthotopic mouse models (Fig. 2A). We found that anti-SIRPα therapy effectively blocked the SIRPα expression in CD45^+^CD11b^+^ TIMs in the tumor (Fig. 2B). Our data also suggested that anti-SIRPα antibody significantly inhibited tumor growth in the subcutaneous tumor model (Fig. 2C–E). As the two major subpopulations of immunosuppressive myeloid cells, TAMs (CD11b^+^F4/80^+^ in mouse) and G-MDSCs (CD11b^+^Ly6G^+^Ly6C^low^ in mouse) are known to impair the antitumor immune response of CD8^+^ T cells [19]. We found that SIRPα blockade significantly elevated the infiltration and function of CD8^+^ T cells while decreasing the infiltrated macrophages and G-MDCSs (Fig. 2F–H and Fig. S2A). Multiplex immunofluorescence (mIF) assay also suggested that anti-SIRPα treatment effectively decreased the infiltration of CD11b^+^ myeloid cells while elevating the infiltration and granzyme B (GZMB) expression of CD8^+^ T cells (Fig. 2I, J). Similarly, in the orthotopic tumor model, we found that anti-SIRPα therapy inhibited the tumor growth and prolonged the survival of mice (Fig. 2K–N). Through mIF and flow cytometry analysis, we found that SIRPα blockade significantly downregulated the infiltrated CD11b^+^ myeloid cells while promoting the infiltration and cytotoxic function of CD8^+^ T cells (Fig. 2O, P). Collectively, these data suggested that SIRPα blockade therapy inhibited tumor growth and infiltration of TIMs while upregulating infiltration and cytotoxic function of CD8^+^ T cells.

### SIRPα blockade therapy inhibited migration, polarization and PD-L1 expression of TIMs in vitro

**Fig. 3:**
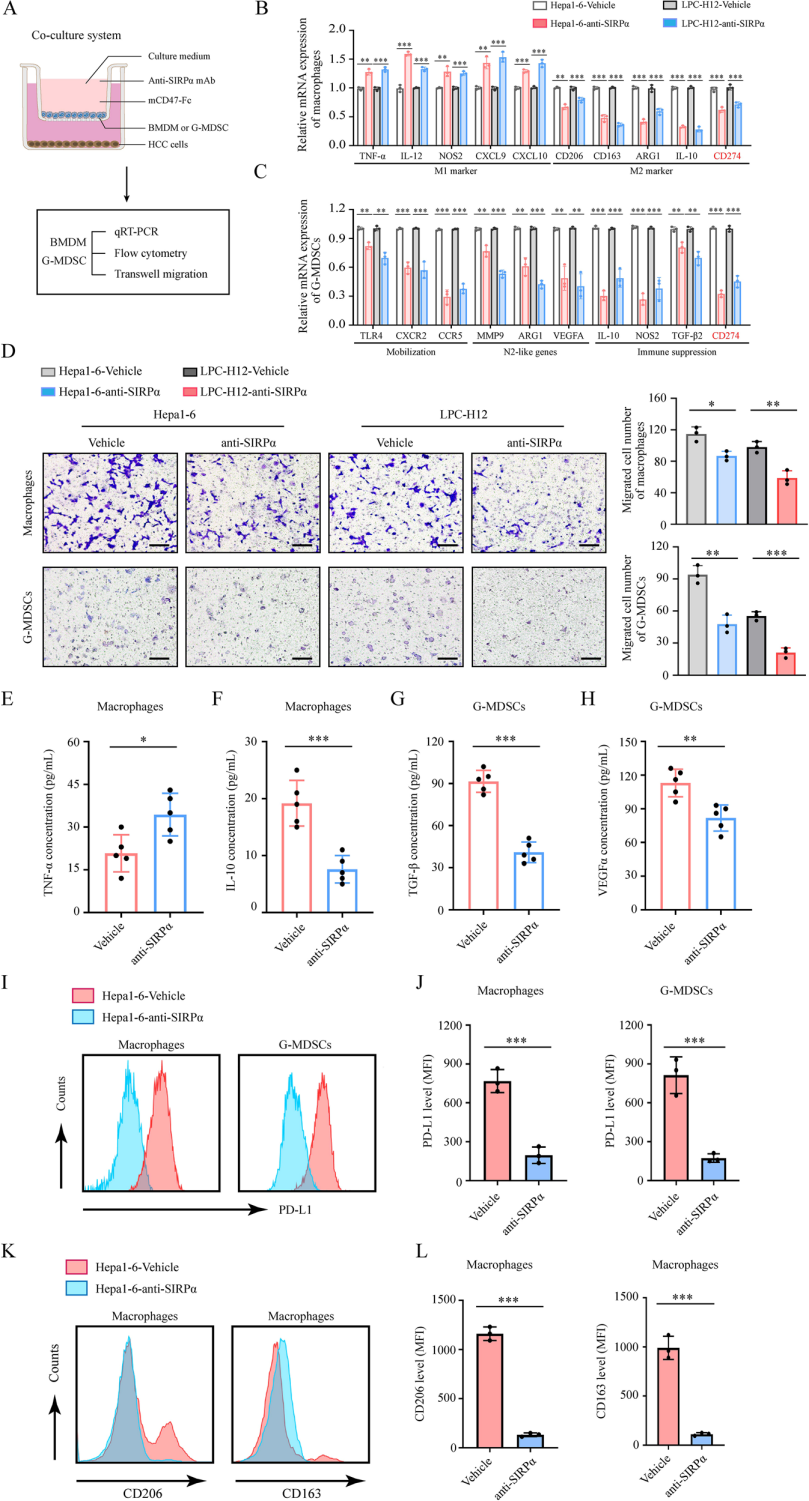
SIRPα blockade suppressed the PD-L1 expression and pro-tumor polarization of TIMs in vitro. **A** Schematic of the in vitro coculture experiments with BMDM or G-MDSC with Hepa1–6 or LPC-H12 cells to mimic liver cancer microenvironment treated with anti-SIRPα mAb (50 ng/ml) or not. BMDM or G-MDSC were incubated with mCD47-Fc for 12 h before analysis. **B**, **C** qRT-PCR analysis of the transcription of indicated markers in BMDM or G-MDSC. **D** Migration of myeloid cells towards the supernatants of the indicated groups. Scale bar, 100 μm. **E**–**H** ELISA assay was used to detect the protein level of TNF-α and IL-10 in the culture medium of BMDM (**E**, **F**) and TGF-β and VEGFα in the culture medium of G-MDSC (**G**, **H**). **I**, **J** Flow cytometry analysis was used to analyze PD-L1 expression in BMDM or G-MDSC after coculture. **K**, **L** M2-polarization markers CD206 or CD163 were detected by flow cytometry in BMDM or G-MDSC after coculture. All data presented are shown as the mean ± SD. *P < 0.05, **P < 0.01, ***P < 0.001, ns not significant.

To further evaluate the potential function of SIRPα blockade in vitro, we established a coculture system to confirm the influence of TME on myeloid cells with or without anti-SIRPα treatment (Fig. 3A). We cocultured murine liver cancer cell lines (Hepa1–6 or LPC-H12) to mimic TME with collected primary bone marrow-derived myeloid cells (BMDM or G-MDSC) (Fig. 3A). Our data suggested that anti-SIRPα inhibited M2 polarization of macrophages and N2-like polarization of G-MDSCs (Fig. 3B, C). Additionally, we found that SIRPα blockade inhibited migration of macrophages and G-MDSCs (Fig. 3D). Furthermore, we found SIRPα blockade increased TNF-α expression of macrophages while decreasing IL-10 expression of macrophages and TGF-β and VEGFα expression of G-MDSCs (Fig. 3E–H). Importantly, consistent with our previous study [15], we found that anti-SIRPα treatment upregulated PD-L1 expression in TIMs, which suggested that SIRPα blockade therapy may inhibit the immune evasion of the tumor (Fig. 3I–L). Above all, these findings suggested that anti-SIRPα treatment suppressed pro-tumor characteristics of M2-like macrophages and G-MDSCs in vitro.

### CD47 blockade inhibited tumor progression in PDX models

**Fig. 4:**
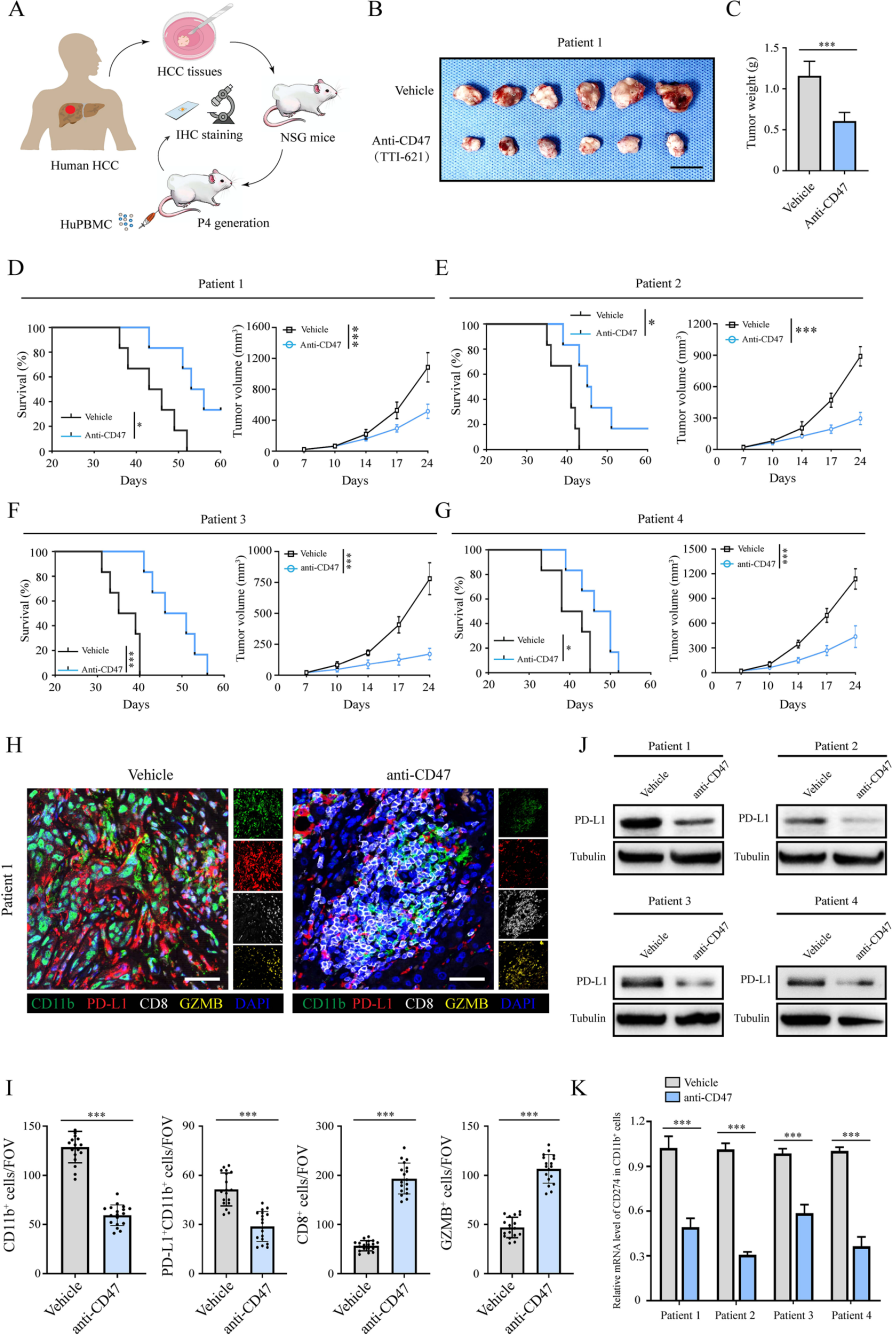
CD47 blockade therapy suppressed tumor progression in the PDX model. **A**–**C** A PDX tumor model was constructed using NSG mice using human HCC tissues. Schematic diagram showing the construction of a PDX tumor model and huPBMC injection through the tail vein. Mice (*n* = 6 for each group) were treated with anti-CD47 antagonist TTI-621 (8 mg/kg, three times a week for 3–4 weeks, i.p.) or vehicle (**A**). The tumor images (scale bar, 1 cm) (**B**), tumor weight (**C**) in the PDX tumor model using HCC tissues from patient 1. **D**–**G** The survival and tumor sizes of the tumor-bearing mice were recorded from patients 1–4. **H**, **I** mIF analysis of the tumor tissues in PDX model was used to detect the TIMs (CD11b), PD-L1, CD8^+^ T cells, and cytotoxic function (GZMB). Scale bar, 50 μm. **J**, **K** Western blot and qRT-PCR analysis were used to detect the PD-L1 expression in TIMs of tumor tissues in the PDX model. TIMs were obtained through magnetic bead sorting. All data presented are shown as the mean ± SD. *P < 0.05, **P < 0.01, ***P < 0.001, ns not significant.

One way of inhibiting the function of TIMs is by blocking signaling through the CD47/SIRPα axis. To study the effect of blocking the CD47/SIRPα axis in treating human HCC, we established a PDX model in mice with or without CD47 blockade treatment using CD47 antagonist TTI-621 (Fig. 4A). We found that anti-CD47 treatment attenuated the tumor growth and prolonged the survival of mice (Fig. 4B–G). Furthermore, we detected the infiltrated CD8^+^ T cells and TIMs in these tumor tissues. We observed that CD47 blockade treatment decreased infiltration of PD-L1^+^CD11b^+^ TIMs while upregulating the infiltration and cytotoxic function of CD8^+^ T cells (Fig. 4H, I). Next, we separated and detected the PD-L1 expression of CD11b^+^ TIMs in these HCC tumors and found that CD47 blockade significantly decreased the PD-L1 expression in TIMs in HCC (Fig. 4J, K). These data suggested that CD47 blockade inhibited HCC growth, infiltration of PD-L1^+^CD11b^+^ TIMs and upregulated infiltration and cytotoxic function of CD8^+^ T cells in the PDX model.

### SIRPα blockade therapy hampered HCC progression and PD-L1 expression of TIMs through downregulating PI3K/AKT signaling in TIMs

**Fig. 5:**
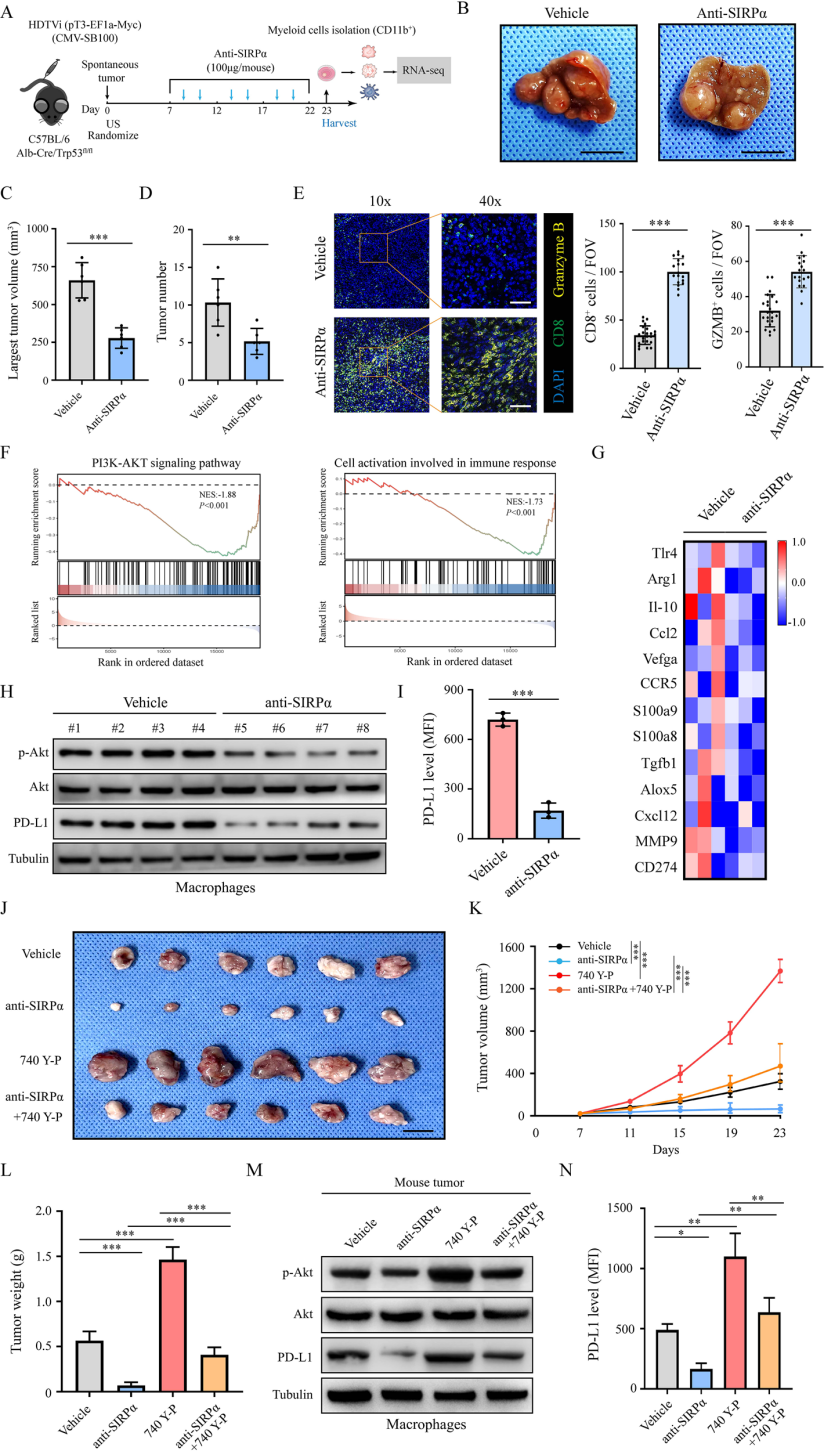
Anti-SIRPα therapy inhibited PI3K/AKT signaling in myeloid cells to suppress tumor progression. **A** A schematic showing the treatment plan in the spontaneous HCC model. The Alb-Cre/Trp53^fl/fl^ mouse model was used to establish a spontaneous HCC model through HDTVi. An ultrasound imaging platform was used to randomize mouse into two groups. Mice (*n* = 6 for each group) were treated with anti-SIRPα mAb or IgG (100 μg/mouse, i.p.). At the endpoint, mice were sacrificed, and the gross appearance of the liver was presented (Scale bar, 1 cm) (**B**), the largest tumor volume (**C**) and tumor number (**D**) was analyzed. **E** The tumor tissues were analyzed by IF to evaluate the infiltration and function of CD8^+^ T cells and GZMB. Scale bar, 50 μm. **F** RNA-seq analysis of the TIMs in spontaneous tumor tissues. GSEA analysis showed that “PI3K/AKT signaling pathway” and “cell activation involved in immune response” were downregulated with anti-SIRPα mAb compared with the control group. **G** Heatmap of genes involved in M2-like phenotype in macrophages or pro-tumor phenotype in G-MDCSs in anti-SIRPα treatment group compared with the control. **H** Western blot analysis was used to detect the expression of PD-L1 and activation of PI3K/AKT signaling in extracted macrophages in the tumor tissues in the indicated group. **I** Flow cytometry analysis of the PD-L1 expression of these macrophages. **J** 740 Y-P (selective agonist of PI3K, 100 mg/kg/day, i.p.) and/or anti-SIRPα mAb was used to treat Hepa1–6 subcutaneous tumor model in mice (*n* = 6 for each group). At the endpoint, mice were sacrificed and tumors were imaged (Scale bar, 1 cm). Tumor growth curves (**K**) and tumor weights (**L**) were analyzed. **M** Macrophages were isolated from each group, and western blot analysis were used to detect the activation of PI3K/AKT signaling and PD-L1 expression. **N** Flow cytometry analysis of the PD-L1 expression of these macrophages in each group. All data presented are shown as the mean ± SD. *P < 0.05, **P < 0.01, ***P < 0.001, ns not significant.

To delineate the underlying mechanism whereby anti-SIRPα inhibited HCC progression in TME, we established a spontaneous tumor model and used anti-SIRPα mAb to treat the tumor (Fig. 5A). After treatment, CD45^+^CD11b^+^ TIMs were isolated from tumor tissues and analyzed by RNA-seq (Fig. 5A). Our results showed that anti-SIRPα mAb effectively inhibited the progression of the spontaneous tumor (Fig. 5B–D). Using IF analysis, we found anti-SIRPα treatment significantly upregulated infiltration and GZMB expression of CD8^+^ T cells in these tumor tissues (Fig. 5E). Through RNA-seq analysis, we detected the transcriptional alterations of CD11b^+^ TIMs in TME treated by anti-SIRPα antibody compared with the control. Gene set enrichment analysis (GSEA) showed that PI3K/AKT signaling and “cell activation involved in immune response” were downregulated in the anti-SIRPα treatment group (Fig. 5F). Additionally, SIRPα blockade decreased the M2-like polarization markers of macrophages and immunosuppressive markers of G-MDSCs (Fig. 5G). We next examined the PD-L1 expression and PI3K/AKT signaling activation in macrophages and G-MDSCs in these tumor tissues, and found that compared with the control group, anti-SIRPα treatment downregulated p-AKT and PD-L1 expression in myeloid cells (Fig. 5H, I and Fig. S3A, B).

Furthermore, we established the Hepa1–6 subcutaneous tumor model and treated the tumor with or without anti-SIRPα antibody or PI3K/AKT signaling agonist 740 Y-P. Our data showed that 740 Y-P reversed the tumor inhibitory function of anti-SIRPα antibody (Fig. 5J–L). Additionally, we found that anti-SIRPα treatment decreased the p-AKT and PD-L1 expression while 740 Y-P reversed this trend in TIMs (Fig. 5M, N and Fig. S3C, D). Herein, these data suggested that SIRPα blockade treatment inhibited HCC growth and PD-L1 expression of TIMs through downregulating PI3K/AKT signaling.

### SIRPα blockade therapy inhibited SIRPα/CD47 axis-mediated PI3K/AKT signaling to suppress migration, PD-L1 expression of myeloid cells in vitro and HCC progression

**Fig. 6:**
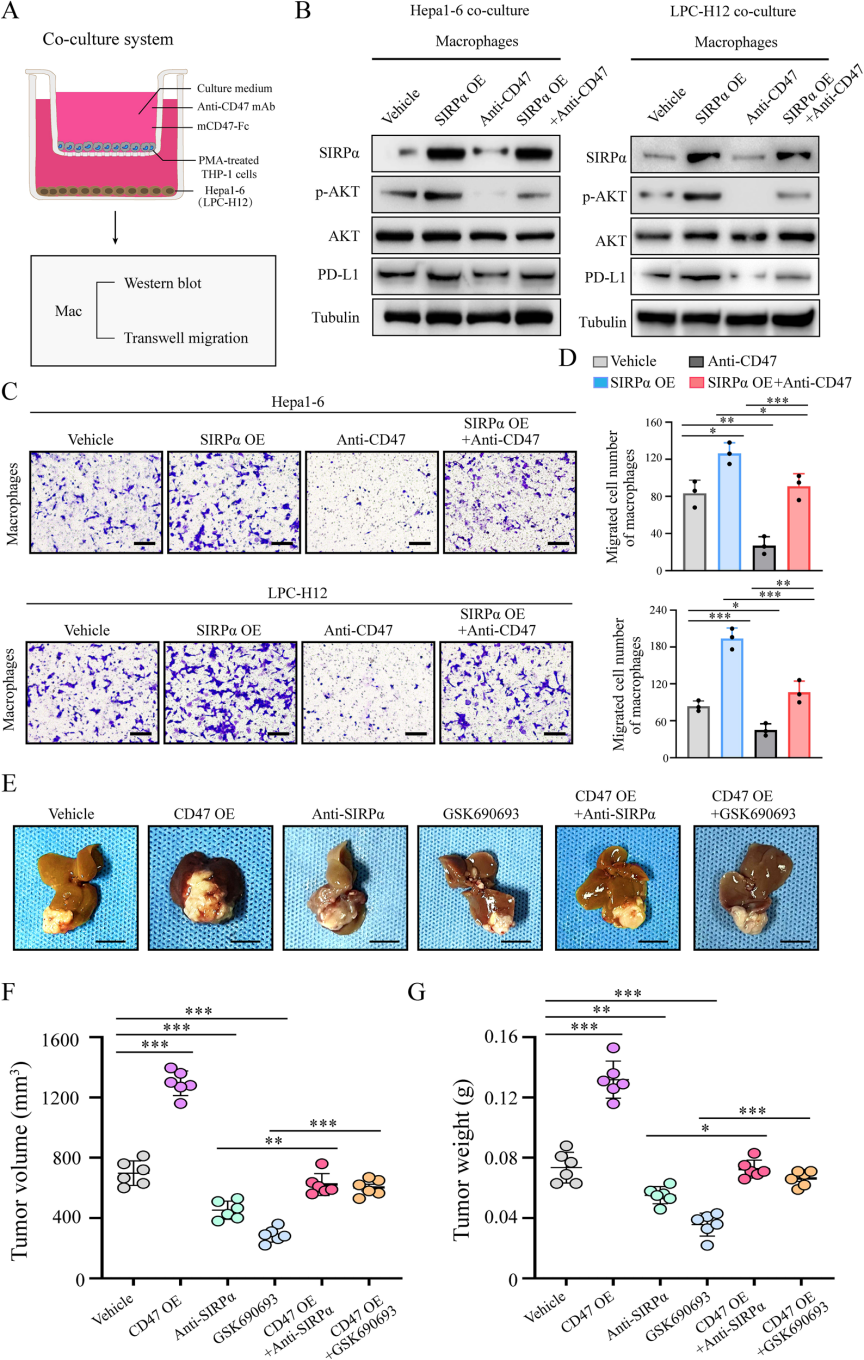
SIRPα/CD47 axis promoted migration, PD-L1 expression of macrophages and HCC growth via upregulating PI3K/AKT signaling. **A** Schematic of the in vitro coculture experiments with PMA-treated THP-1 cells cocultured with Hepa1–6 or LPC-H12 cells to mimic liver cancer microenvironment treated with anti-CD47 mAb (10 μg/ml) or not. Macrophages were incubated with mCD47-Fc for 12 h before analysis. **B** Western blot analysis was used to detect the expression of indicated proteins in macrophages. **C**, **D** Transwell assay was used to detect the migration of macrophages towards the supernatants of the indicated groups. Scale bar, 100 μm. **E** Orthotopic HCC model using Hepa1–6 was established. Wild-type C57BL/6 mice (*n* = 6 for each group) were treated with anti-SIRPα mAb (100 μg/mouse, i.p.) and/or GSK690693 (30 mg/kg/day, i.p.) compared with the control group. Representative livers in each group were photographed at the endpoint (Scale bar, 1 cm). **F**, **G** Statistical analysis of tumor volume and tumor weight of the mice. All data presented are shown as the mean ± SD. *P < 0.05, **P < 0.01, ***P < 0.001, ns not significant.

To further determine the essential role of SIRPα/CD47 axis in mediating PD-L1 expression and migration of macrophages through PI3K/AKT signaling activation, we first used a coculture system to assess the trafficking of macrophages to HCC cells (Fig. 6A). The THP-1 cell line stably overexpressing SIRPα was constructed and cocultured with Hepa1–6 or LPC-H12 cells (Fig. 6A). We observed that SIRPα overexpression in macrophages activated PI3K/AKT signaling, PD-L1 expression and migration, while anti-CD47 antibody reversed this trend (Fig. 6B–D). Furthermore, we constructed an orthotopic mouse model using Hepa1–6 cells stably overexpressing CD47 compared with the control (Fig. 6E–G). We observed that CD47 overexpression promoted the growth of HCC cells while anti-SIRPα antibody or PI3K/AKT signaling inhibitor GSK690693 effectively reversed this trend (Fig. 6E–G). Through mIF and flow cytometry, we found that tumor intrinsic CD47 overexpression inhibited the infiltration and cytotoxic function of CD8^+^ T cells, while upregulating the infiltration of myeloid cells compared with the control (Fig. S4A–I). In contrast, anti-SIRPα antibody or GSK690693 significantly reversed this trend (Fig. S4A–I). These data suggested that SIRPα/CD47 axis activation promotes PD-L1 expression, migration of macrophages and HCC progression through upregulating PI3K/AKT signaling.

### SIRPα blockade therapy potentiates anti-PD-L1 therapy in treating HCC

**Fig. 7:**
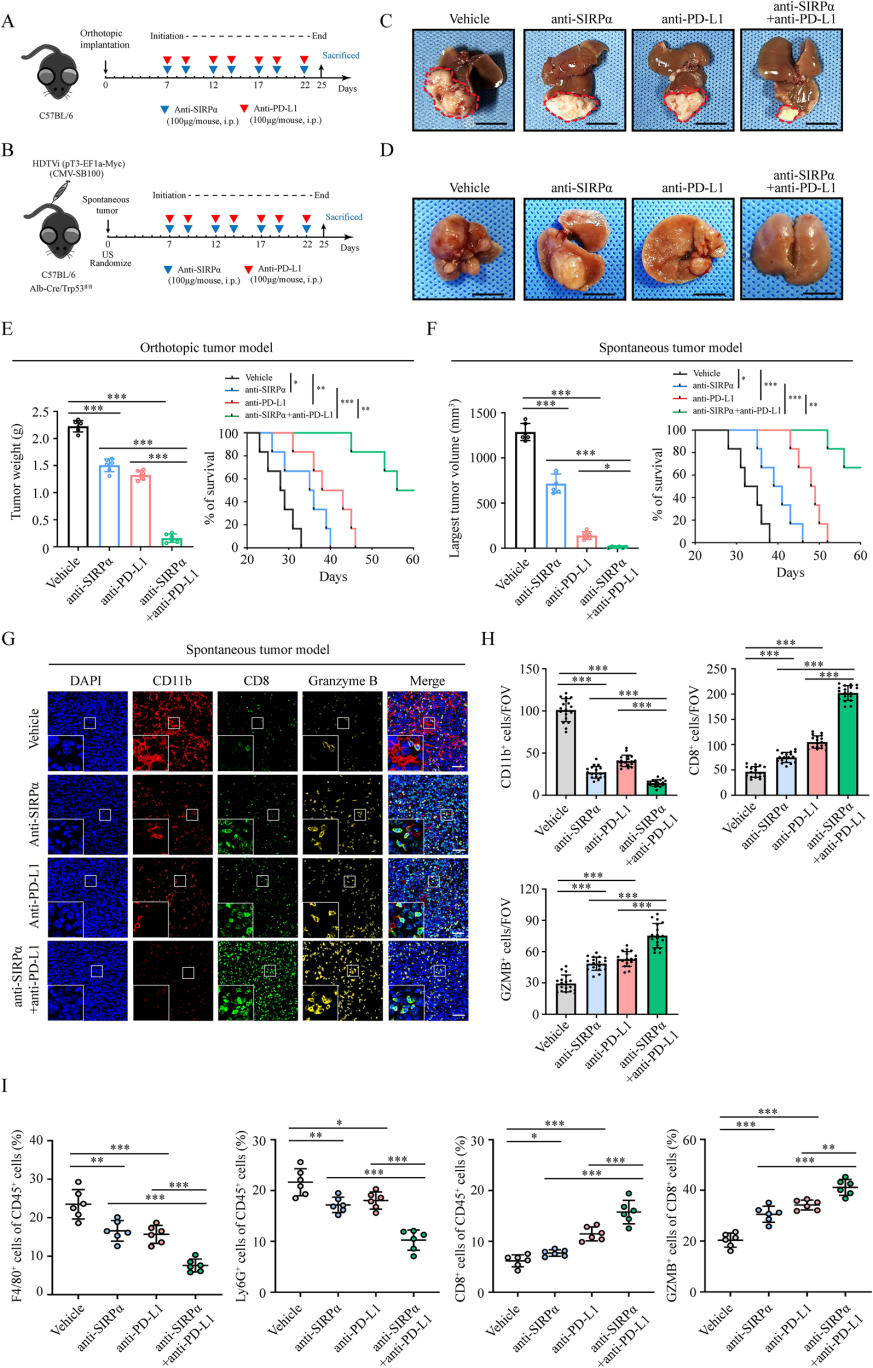
SIRPα blockade therapy effectively enhanced anti-PD-L1 therapy in orthotopic and spontaneous mouse models. **A**, **B**) Schematics showing the treatment plan in the Hepa1–6 orthotopic and spontaneous HCC model. C57BL/6 mice (*n* = 6 for each group) were treated with anti-SIRPα mAb (100 μg/mouse, i.p.) and/or anti-PD-L1 mAb (100 μg/mouse, i.p.) compared with the IgG group. **C**, **D** Representative livers in each group were photographed at the endpoint (Scale bar, 1 cm). **E**, **F** Tumor weight and survival of the mice were analyzed in the indicated group. **G**, **H** In the spontaneous tumor model, mIF analysis was used to detect the expression of TIMs and CD8^+^ T cells. Scale bar, 50 μm. **I** Flow cytometry analysis was used to evaluate the macrophages, G-MDSCs, CD8^+^ T cells and GZMB in tumor tissues. All data presented are shown as the mean ± SD. *P < 0.05, **P < 0.01, ***P < 0.001, ns not significant.

To investigate whether anti-SIRPα treatment could synergize with anti-PD-L1 therapy, we established orthotopic and spontaneous tumor models (Fig. 7A, B). Our data suggested that anti-SIRPα combined with anti-PD-L1 therapy effectively inhibited the progression of HCC and significantly prolonged the survival of mice (Fig. 7C–F). We next examined the infiltration of TIMs and CD8^+^ T cells in these tumor tissues using mIF and flow cytometry. Our results showed that compared with the monotherapy group, combination therapy using anti-SIRPα and anti-PD-L1 antibody significantly decreased the infiltration of TIMs, whereas it increased the infiltration and cytotoxic function of CD8^+^ T cells (Fig. 7G–I and Fig. S5A, B). Collectively, these data showed that anti-SIRPα treatment could synergize with anti-PD-L1 therapy and effectively promote the therapeutic efficacy of immunotherapy in treating HCC.

## Discussion

SIRPα is widely studied as an inhibitory receptor on macrophages, which recognizes as a ligand of the cell surface molecule CD47, expressed mainly on myeloid cells and also upregulated on cancer cells [11, 20, 21]. Pharmacological blockade of CD47 or SIRPα is a promising anticancer therapy. However, more studies have been focused on the CD47 blockade therapy, although it has been associated with multiple side effects, including circulating red blood cells (RBCs) toxicity [21]. In our study, we first investigated the function of anti-SIRPα therapy plus immunotherapy in treating HCC in a spontaneous mouse model, which is more comprehensive to verify the synergistic effect of anti-SIRPα therapy and anti-PD-L1 therapy combination therapy. Our findings suggested that SIRPα blocked therapy inhibited the immunosuppressive TME in HCC through targeting TIMs, which effectively synergize with anti-PD-L1 therapy in treating HCC.

Myeloid cells are the main component of the TME and play crucial roles in regulating tumor progression and metastasis [7]. Accumulating evidence showed that myeloid inhibitors can decrease the immunosuppressive effect of the TME and promote chemotherapy or immunotherapy responses [10, 22, 23]. In this study, we found that the proportion of MDSCs plus macrophages are the main components of TIMs. Furthermore, SIRPα was highly expressed in macrophages and MDSCs. A recent study suggested that SIRPα^+^ macrophages infiltration was elevated in nonalcoholic steatohepatitis (NASH), and anti-SIRPα promoted necroptotic hepatocytes engulfment and inhibited hepatic fibrosis [24]. Another study also suggested that radiotherapy combined with SIRPα and PD-1 blockade significantly promoted systemic tumor regressions [25]. Here, we found that anti-SIRPα therapy significantly inhibited tumor growth through hampering the immunosuppressive TME in mouse. Moreover, in the PDX model, we observed that SIRPα blockade therapy effectively inhibited tumor growth using HCC tissues from patients.

In the TME of liver cancer, myeloid cells can be recruited and polarized to tumor-promoting M2-TAMs or G-MDSCs [5, 8]. Here, we found that the TME of liver cancer has the capacity to polarize myeloid cells to a pro-tumor status. Additionally, compared with the control group, SIRPα blockade therapy significantly inhibited the PD-L1 expression and polarization of myeloid cells to a pro-tumor status. In the PDX model, we further confirmed that CD47/ SIRPα axis blockade effectively downregulated PD-L1 expression in TIMs. Our previous study showed that PD-L1^+^ tumor-associated neutrophils play an essential role in promoting an immunosuppressive TME and immunotherapy resistance [26]. Additionally, increasing evidence suggested the immunosuppressive and pro-tumor role of PD-L1^+^ myeloid cells in cancer [27–29]. In this study, we revealed that SIRPα blockade therapy could inhibit PD-L1^+^ myeloid cells in TME to hamper immunosuppression and progression of HCC.

PI3K is expressed in immune cells and is activated by G protein-coupled receptors. PI3K/AKT signaling plays a pivotal role in macrophage differentiation and neutrophil activation [5, 30]. In our study, we found that SIRPα blockade therapy inhibited PI3K/AKT signaling in myeloid cells to suppress the PD-L1 expression and polarization of TIMs to pro-tumor M2-like TAMs or G-MDSCs. However, the PI3K/AKT signaling influences TIMs' function in the TME and needs further exploration.

Based on the role of the CD47-SIRPα axis as a regulator of tumor cell fate, a series of molecules that block the CD47-SIRPα axis are currently under clinical development for tumor indications, and in some cases have encouraging clinical outcomes [5]. Immunotherapy methods that block the CD47-SIRPα signaling axis have shown promising preclinical activity, significantly inducing phagocytosis in various types of cancer [31, 32]. However, CD47-SIRPα blockade has encountered several challenges in clinical applications, including a lack of therapeutic efficacy and toxicity [33]. SIRPα is mainly expressed on a limited number of cell types, and targeting the CD47-SIRPα axis using SIRPα inhibitors seems to be better than CD47 blockade, which might decrease the side effects of anti-CD47 therapy [33]. Here, we found that anti-SIRPα treatment could effectively synergize with anti-PD-L1 therapy in treating HCC in vivo without significant changes in body weight or serum indicators of liver and kidney function in mice. Our findings may shed light on the therapeutic strategies in treating HCC using anti-SIRPα plus anti-PD-L1 combination therapy.

The current state of CD47-SIRPα blocking therapy have some limitations, including circulating RBC toxicity. To push the clinical application of CD47-SIRPα axis blockade therapy forward, it is important to evaluate the crosstalk between immune cells and tumors in TME. Our future study will focus on the new mechanisms whereby CD47-SIRPα blocking therapy impact myeloid cells in TME.

## Conclusion

**Fig. 8:**
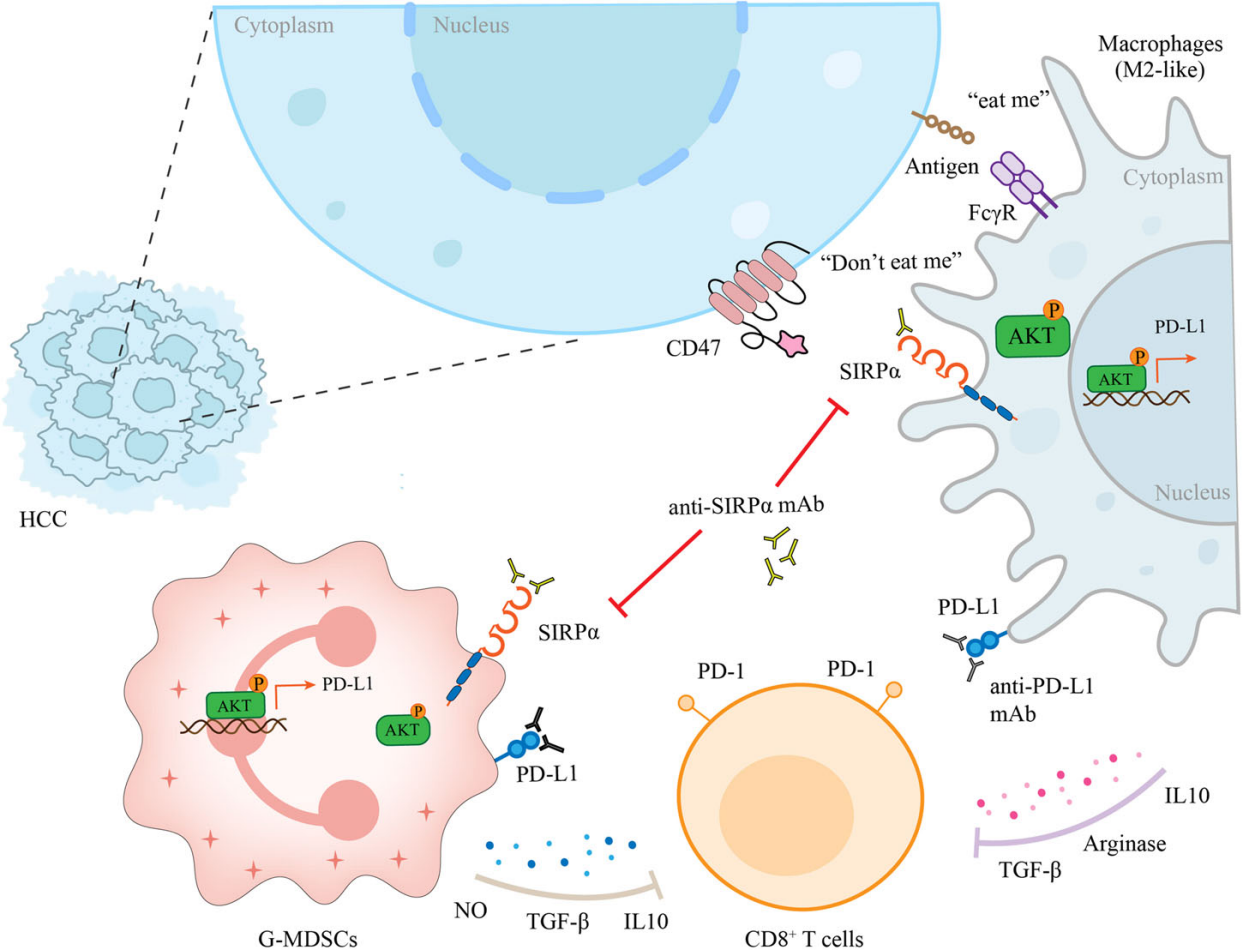
A schematic illustration that anti-SIRPα inhibited the immunosuppressive function and PD-L1 expression of TIMs through downregulating PI3K/AKT signaling in myeloid cells.

SIRPα-blockade therapy reversed the immunosuppressive TME through inhibiting the pro-tumor polarization of PD-L1^+^ TIMs. Anti-SIRPα plus anti-PD-L1 combination therapy could effectively inhibit HCC progression (Fig. 8).

## Availability of data and material

The data used to support the findings of this study are included and available within the article.

## Supplementary information

Supplementary materials

Supplementary Table 1

Supplementary Table 2

original data
