# Supplementary material for: SIRPα blockade therapy potentiates immunotherapy by inhibiting PD-L1+ myeloid cells in hepatocellular carcinoma
Source: Cell Death Dis. 2025 Jun 16;16(1):451. doi: 10.1038/s41419-025-07779-7 (PMC12170831; doi:10.1038/s41419-025-07779-7)
Supplement: Supplementary file 1 — Supplementary materials [file 41419_2025_7779_MOESM1_ESM.docx]

**Cell lines and cell culture**

The mouse liver cancer cell line Hepa1-6 was obtained from the Liver Cancer Institute, Fudan University (Shanghai, China), the mouse liver cancer cell lines LPC-H12 and H22 were obtained from the Cell Bank of Shanghai Institute for Biological Sciences (Chinese Academy of Science, Shanghai, China). Dulbecco’s modified Eagle’s medium (DMEM, Gibco, USA) or RPMI 1640 medium (Gibco) supplemented with 10% fetal bovine serum (FBS, Gibco) were used to culture cells. 100 U/L penicillin/streptomycin (Gibco) was added in culture medium in a humidified incubator containing 5% CO_2_ at 37 °C.

**Quantitative Real-Time Polymerase Chain Reaction**

Total RNA was extracted through RNAiso Plus (9109, Takara) according to the manufacturer’s instructions. The RT reagent Kit with gDNA Eraser (RR047, Takara) was utilized for RNA reverse transcription. A LightCycler 480 (Roche Diagnostics, Germany) was used for Quantitative real-time PCR and data was normalized using internal control GAPDH. ΔΔCt method was used to calculate results and the primers used were listed in Table S2.

**Western blot analysis**

Western blot analysis of target proteins was performed as previously described ^[1]^. Bands visualization and intensity quantitation for western blot were performed using an electrogenerated chemiluminescence (ECL) imaging system (Tanon, Shanghai, China). The proteins were probed with the indicated primary antibody: anti-PD-L1 (Abcam, ab237726, 1:1000), anti-PD-L1 (Abcam, ab213480, 1:1000), anti-phospho-AKT (Cell Signaling Technology, #9271, 1:1000), anti-AKT (Cell Signaling Technology, #9272, 1:1000), anti-Tubulin (Abcam, ab7291, 1:1000).

**Multiplex IF assay**

Slides were deparaffinized and rehydrated in xylene and graded ethanol. Citrate buffer was used to retrieve antigen and tissues were blocked by 5% BSA. Primary antibodies were employed to incubate tissues overnight at 4℃. Primary antibodies include anti-CD11b (Abcam, ab133357, 1:200), anti-SIRPα (Abcam, ab191419, 1:200), anti-CD8 (Abcam, ab237709, 1:200), anti-CD8 (Abcam, ab217344, 1:200), anti-PD-L1 (Abcam, ab237726, 1:200), anti-PD-L1 (Abcam, ab213480, 1:200), anti-Granzyme B (Abcam, ab255598, 1:100).

**Flow cytometry**

Tissues were treated by mechanical and enzymatic disruption in Hanks balanced salt solution (HBSS) with 1mg/ml collagenase P (Roche, Germany) using the gentleMACS™ Dissociator (Miltenyi Biotec, Auburn, CA). Digestion was terminated through 1% BSA diluted in cold PBS and 70-μm strainers (Beyotime) was used to filter single-cell suspensions. Dead Cell Stain was used to distinguish dead cells, and live cells were further incubated with FC Block (BD Pharmingen, Clone 2.4G2) to block cells. FACS Aria III cytometer (BD biosciences, USA) was used to perform flow cytometry analysis and cell separation. FlowJo software (v 10.4) was used to analyze data. Fluorochrome-conjugated antibodies include PE-SIRPα, PerCP-Cy5.5-CD45, PerCP-Cy5.5-CD3, FITC-CD8, BV421-Granzyme B, APC-CD11b and FITC-PD-L1 were purchased from BioLegend (San Diego, CA, USA).

***In vitro* myeloid cells isolation and treatment**

Bone marrow-derived myeloid cells (BMDM and G-MDSC) were isolated by pestling the femurs of 8-week-old WT C57BL/6 mice. RBCs were removed and IMDM media supplemented with 10% FBS and 20 ng/ml M-CSF was used to culture BMDM for 7 days. 1640 culture medium containing 10% FBS, 40 ng/ml GM-CSF, and 20 ng/ml G-CSF was used to incubate G-MDSC.

**Supplemental Figures**


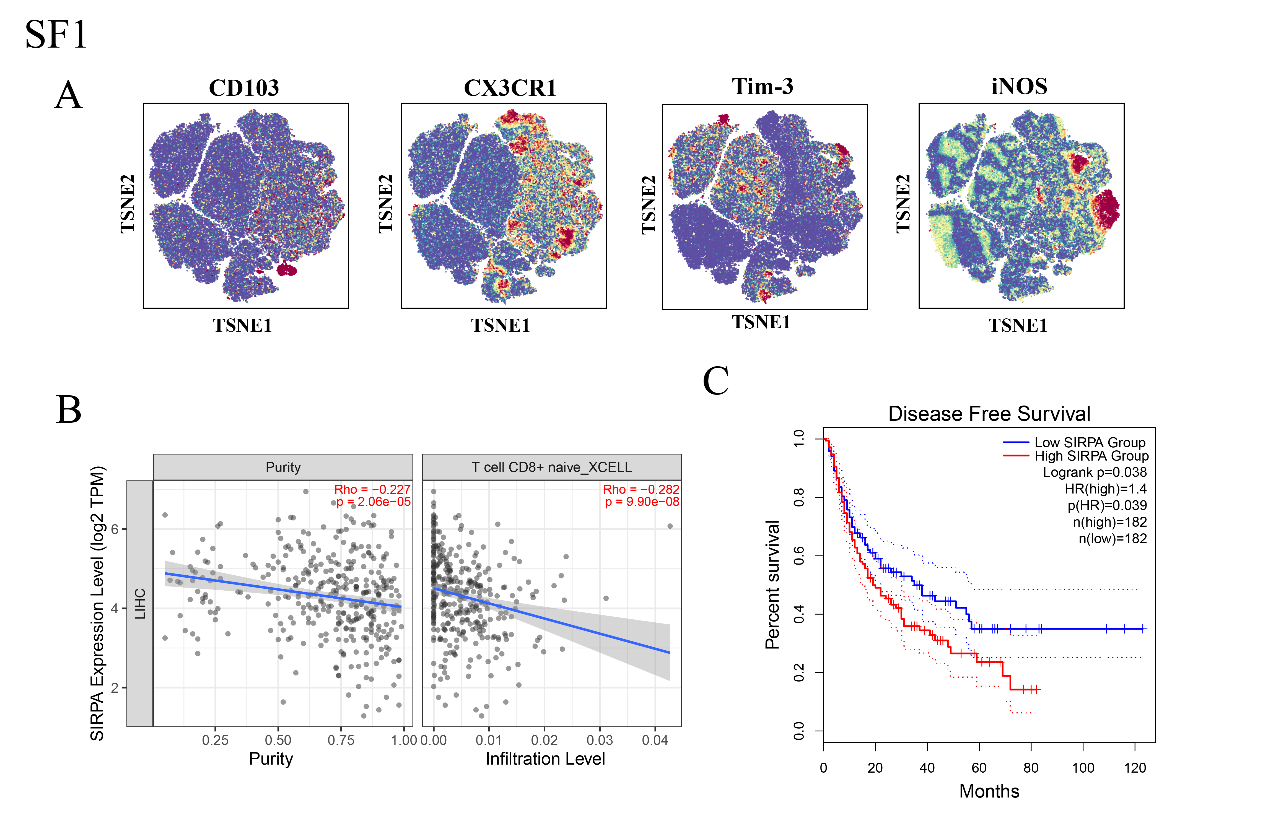


**Figure S1. SIRPα expression was negatively correlated with CD8^+^ T cells infiltration in HCC and poor prognosis of HCC patients**

(A) t-SNE plot of all myeloid cells colored according to the expression levels of CD103, CX3CR1, Tim-3, iNOS; (B) Correlation between SIRPA expression and CD8^+^ T cells infiltration in HCC analyzed by Timer 2.0 data base. (C) Disease free survival of HCC patients in TCGA database with high or low SIRPα expression. * *P* < 0.05, ** *P* < 0.01, *** *P* < 0.001, ns: not significant.


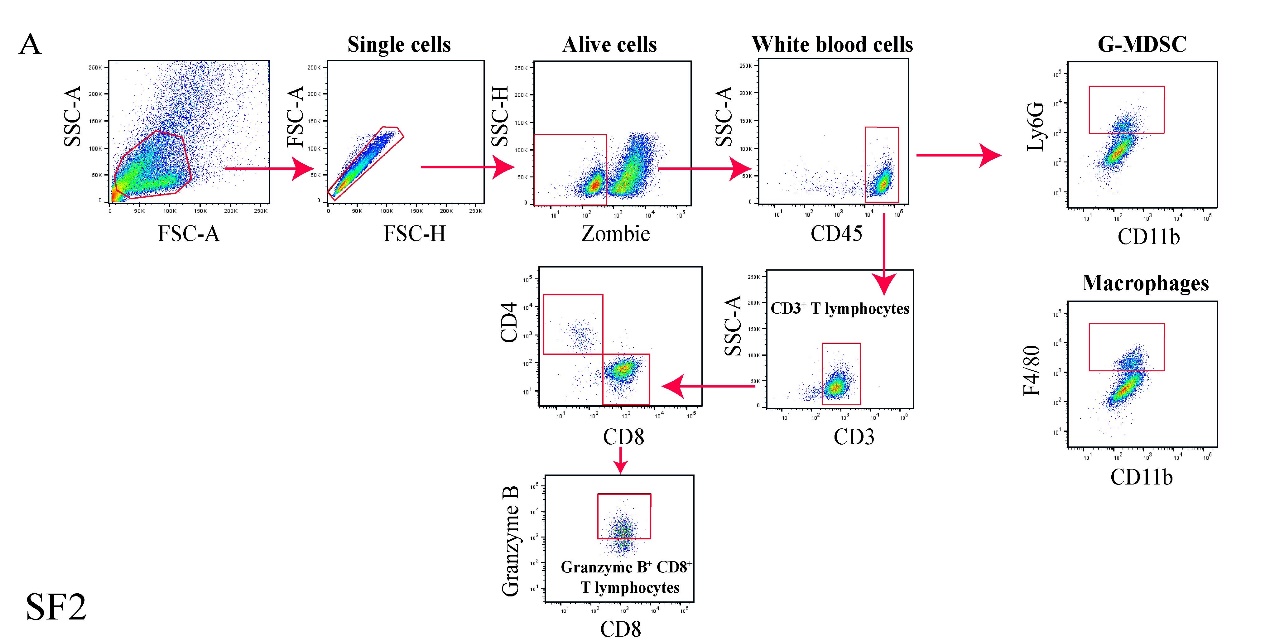


**Figure S2. Gating strategies to identify immune cells from mouse HCC tissues.** (A) The gating strategies to identify CD8^+^ T cells and myeloid cells (Macrophages or G-MDSCs) in HCC tissues from mouse.


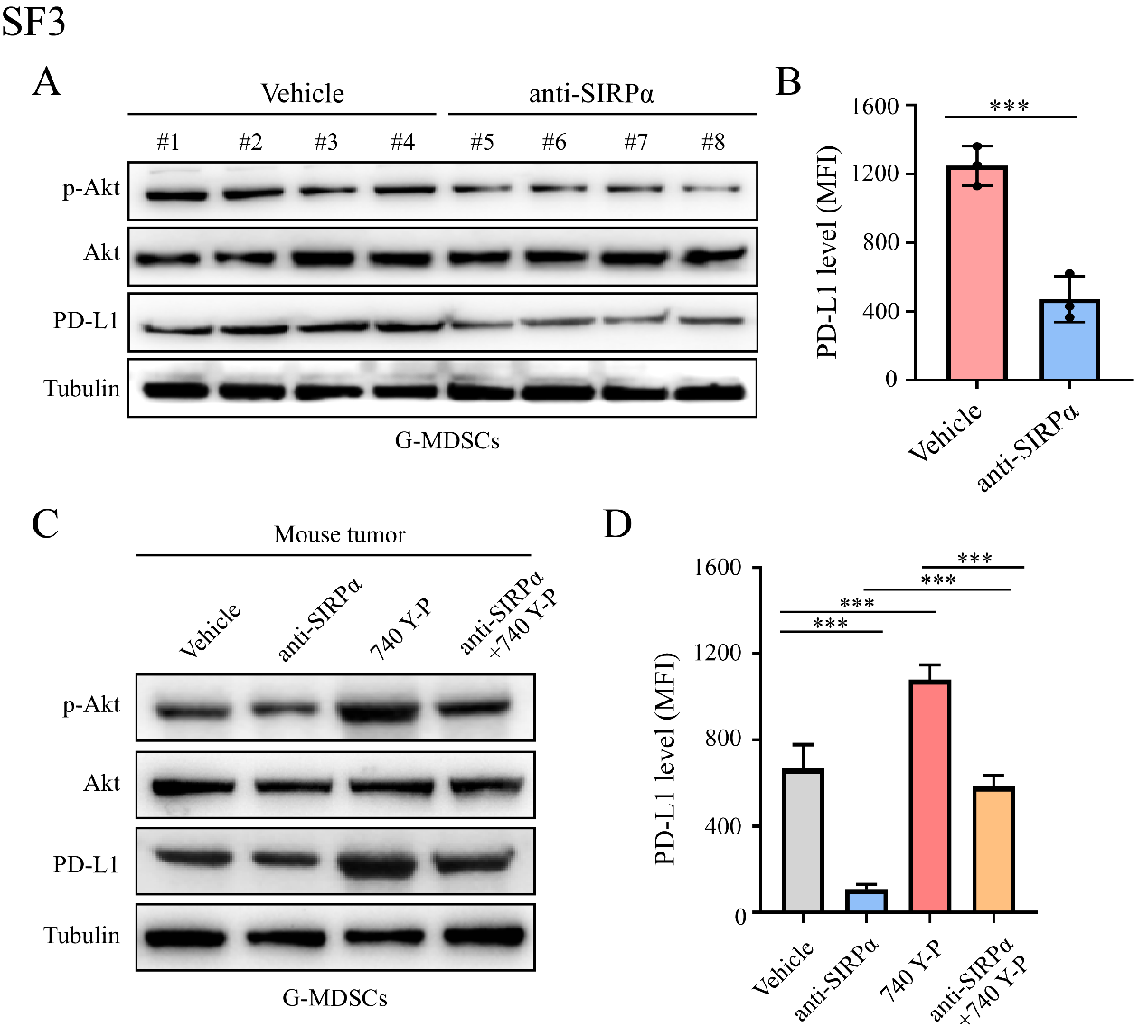


**Figure S3. Anti-SIRPα therapy inhibited PD-L1 expression and PI3K/AKT signaling in G-MDSCs**

(A) Western blot analysis was used to detect the expression of PD-L1 and activation of PI3K/AKT signaling in extracted G-MDSCs in tumor tissues in indicated group. (B) Flow cytometry analysis of the PD-L1 expression of these G-MDSCs. (C) G-MDSCs were isolated from each group and western blot analysis was used to detect the activation of PI3K/AKT signaling and PD-L1 expression. (D) Flow cytometry analysis of the PD-L1 expression of these myeloid cells in each group. * *P* < 0.05, ** *P* < 0.01, *** *P* < 0.001, ns: not significant.


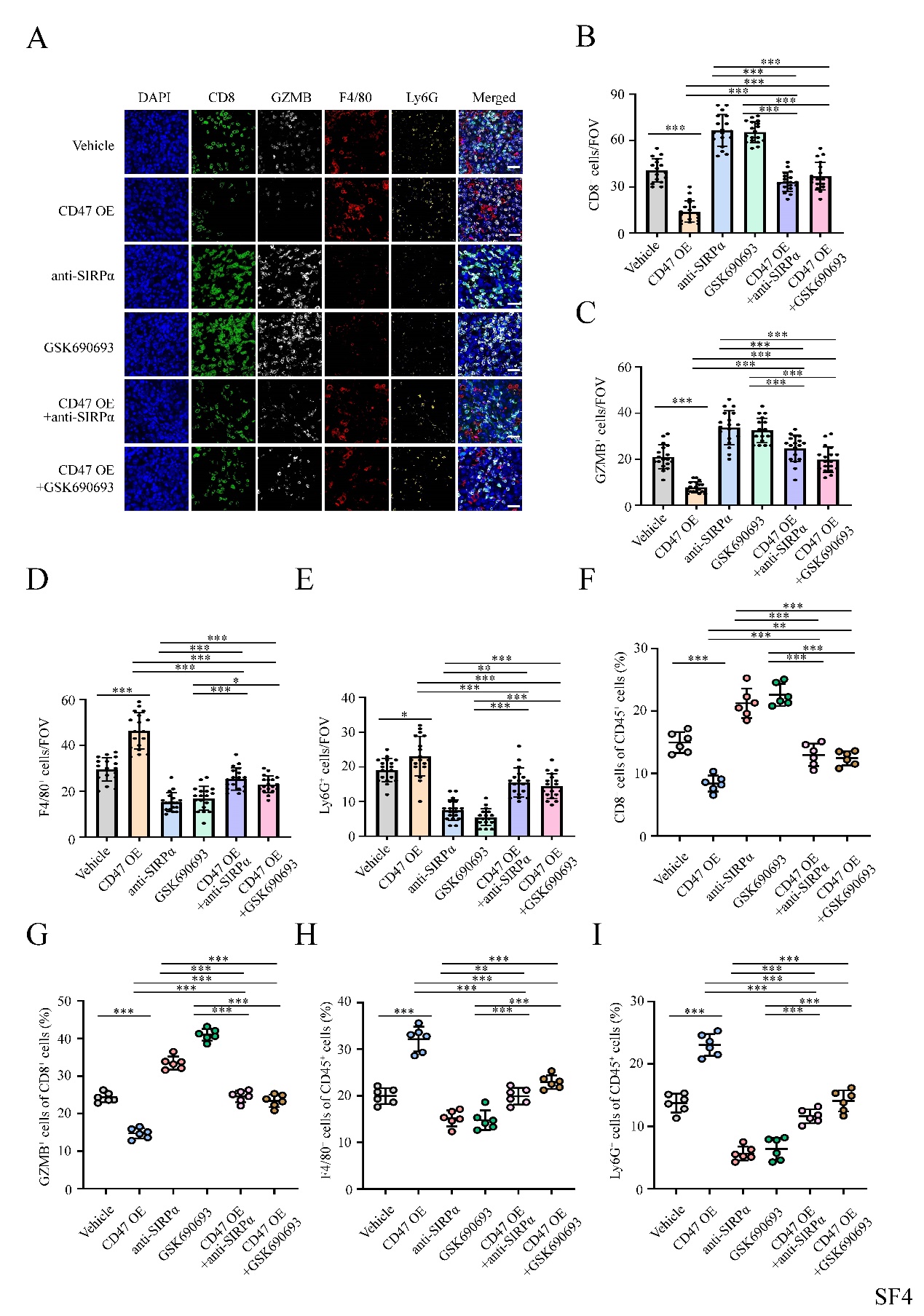


**Figure S4. Anti-SIRPα therapy or GSK690693 inhibited tumor intrinsic CD47 overexpression-mediated immunosuppression of HCC.**

(A-E) In orthotopic tumor model, mIF analysis was used to detect the expression of CD8, GZMB, Ly6G and F4/80 in HCC tissues in indicated groups at the end point of experiments. (G-I) Flow cytometry analysis was used to evaluate the macrophages, G-MDSCs, CD8^+^ T cells and GZMB in tumor tissues. * *P* < 0.05, ** *P* < 0.01, *** *P* < 0.001, ns: not significant.


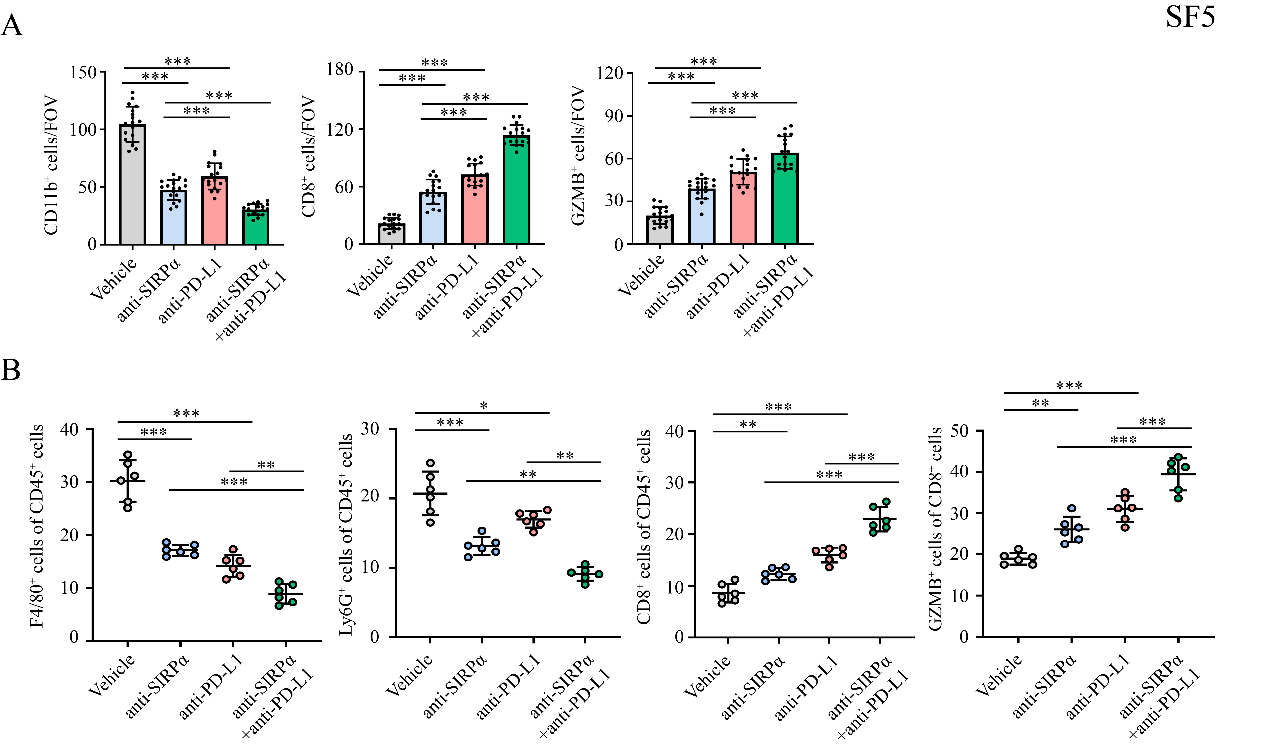


**Figure S5. Anti-SIRPα therapy plus anti-PD-L1 therapy inhibited the immunosuppressive TME**

(A) In orthotopic tumor model, mIF analysis was used to detect the expression of TIMs and CD8^+^ T cells. (B) Flow cytometry analysis was used to evaluate the macrophages, G-MDSCs, CD8^+^ T cells and GZMB in tumor tissues. * *P* < 0.05, ** *P* < 0.01, *** *P* < 0.001, ns: not significant.

**Reference**

[1] Xie P, Guo L, Yu Q, Zhao Y, Yu M, Wang H et al. ACE2 Enhances Sensitivity to PD-L1 Blockade by Inhibiting Macrophage-Induced Immunosuppression and Angiogenesis. *Cancer Res*. 2025;85(2):299-313.
