# Supplementary material for: SIRPα blockade therapy potentiates immunotherapy by inhibiting PD-L1+ myeloid cells in hepatocellular carcinoma
Source: Cell Death Dis. 2025 Jun 16;16(1):451. doi: 10.1038/s41419-025-07779-7 (PMC12170831; doi:10.1038/s41419-025-07779-7)
Supplement: Supplementary file 2 — Supplementary Table 1 [file 41419_2025_7779_MOESM2_ESM.docx]

| **Table S1**-Information of 15 antibodies used in the CyTOF assay. | | | | | | | | | |  |
| --- | --- | --- | --- | --- | --- | --- | --- | --- | --- | --- |
|  |  |  |  |  |  |  |  |  |  |  |
| List | Label |  | Marker |  | Clone |  | Dilution |  | Manufacturer |  |
|  |  |  |  |  |  |  |  |  |  |  |
| 1 | 89Y |  | CD45 |  | 30-F11 |  | 1:400 |  | BioLegend |  |
| 2 | 115In |  | Ki-67 |  | SolA15 |  | 1:200 |  | eBioscience |  |
| 3 | 139La |  | CD172a (SIRPα） |  | P84 |  | 1:100 |  | BioLegend |  |
| 4 | 141Pr |  | CD103 |  | 2E7 |  | 1:50 |  | BioLegend |  |
| 5 | 142Nd |  | MHC II (I-A/I-E) |  | M5/114.15.2 |  | 1:800 |  | BioLegend |  |
| 6 | 144Nd |  | CX3CR1 |  | SA011F11 |  | 1:400 |  | BioLegend |  |
| 7 | 145Nd |  | Ly6C |  | HK1.4 |  | 1:800 |  | BioLegend |  |
| 8 | 146Nd |  | CD206 (MMR) |  | C068C2 |  | 1:50 |  | BioLegend |  |
| 9 | 147Sm |  | CD274 (B7-H1, PD-L1) |  | 29E.2A3 |  | 1:100 |  | BioLegend |  |
| 10 | 148Nd |  | CD11b |  | M1/70 |  | 1:800 |  | BioLegend |  |
| 11 | 149Sm |  | CD11c |  | N418 |  | 1:100 |  | BioLegend |  |
| 12 | 150Nd |  | Ly6G |  | 1A8 |  | 1:800 |  | BioLegend |  |
| 13 | 151Eu |  | F4/80 |  | Cl:A3-1 |  | 1:200 |  | Biorad |  |
| 14 | 152Sm |  | iNOS |  | CXNFT |  | 1:50 |  | eBioscience |  |
| 15 | 153Eu |  | CD366 (Tim-3) |  | RMT3-23 |  | 1:200 |  | BioLegend |  |
