# Supplementary material for: SIRPα blockade therapy potentiates immunotherapy by inhibiting PD-L1+ myeloid cells in hepatocellular carcinoma
Source: Cell Death Dis. 2025 Jun 16;16(1):451. doi: 10.1038/s41419-025-07779-7 (PMC12170831; doi:10.1038/s41419-025-07779-7)
Supplement: Supplementary file 3 — Supplementary Table 2 [file 41419_2025_7779_MOESM3_ESM.docx]

| **Table S2**-Information of primers. | | |  |
| --- | --- | --- | --- |
| Primer name | Primer sequence | |  |
|  |  |  |  |
|  | Forward Reverse | |  |
| Mouse IL-12a | ATGCGTTACAAGCTCAAG | ATGGCTTCAGCTGCAAGTTC |  |
| Mouse TNF-α | CCCTCACACTCAGATCATCTTCT | GCTACGACGTGGGCTACAG |  |
| Mouse NOS2 | GTTCTCAGCCCAACAATACAAGA | GTGGACGGGTCGATGTCAC |  |
| Mouse CXCL9 | GTTCGAGGAACCCTAGTGATAAG | GTTTGAGGTCTTTGAGGGATTTG |  |
| Mouse CXCL10 | TCAGGCTCGTCAGTTCTAAGT | CCTTGGGAAGATGGTGGTTAAG |  |
| Mouse CD206 | CAGGTGTGGGCTCAGGTAGT | TGTGGTGAGCTGAAAGGTGA |  |
| Mouse CD163 | GGTGGACACAGAATGGTTCTTC | CCAGGAGCGTTAGTGACAGC |  |
| Mouse ARG1 | AGGAGCTGTCATTAGGGACATC | CTCCAAGCCAAAGTCCTTAGAG |  |
| Mouse IL-10 | TGCACTACCAAAGCCACAAG | TGATCCTCATGCCAGTCAGT |  |
| Mouse TGF-β2 | CTCGACATGGATCAGTTTATGC | ATAAACCTCCTTGGCGTAGTAC |  |
| Mouse TLR4 | GCCATCATTATGAGTGCCAATT | AGGGATAAGAACGCTGAGAATT |  |
| Mouse VEGFA | GTCCGATTGAGACCCTGGTG | TTGACCCTTTCCCTTTCCTCG |  |
| Mouse MMP9 | CGACGACGACGAGTTGTG | CATGGGGCACCATTTGAGTT |  |
| Mouse CCR5 | TGCTGCCTAAACCCTGTCAT | CGATCAGGATTGTCTTGCTGGA |  |
| Mouse CXCR2 | TGTCTGCTCCCTTCCATCTT | CCATTTCCTCTCCTCCAGCT |  |
| Mouse CD274 | TGAGCAAGTGATTCAGTTTGTG | CATTTCCCTTCAAAAGCTGGTC |  |
| Human CD274 | AGGGCATTCCAGAAAGATGAG | TTGGGAACCGTGACAGTAAAT |  |
| Mouse GAPDH | CGTAGACAAAATGGTGAAGGTCG | CCTTCCACAATGCCAAAGTTGTC |  |
| Human GAPDH | CTGGGCTACACTGAGCACC | AAGTGGTCGTTGAGGGCAATG |  |
