# Supplementary figures and images for: SIRPα blockade therapy potentiates immunotherapy by inhibiting PD-L1+ myeloid cells in hepatocellular carcinoma
Source: Cell Death Dis. 2025 Jun 16;16(1):451. doi: 10.1038/s41419-025-07779-7 (PMC12170831; doi:10.1038/s41419-025-07779-7)

Figure 5H

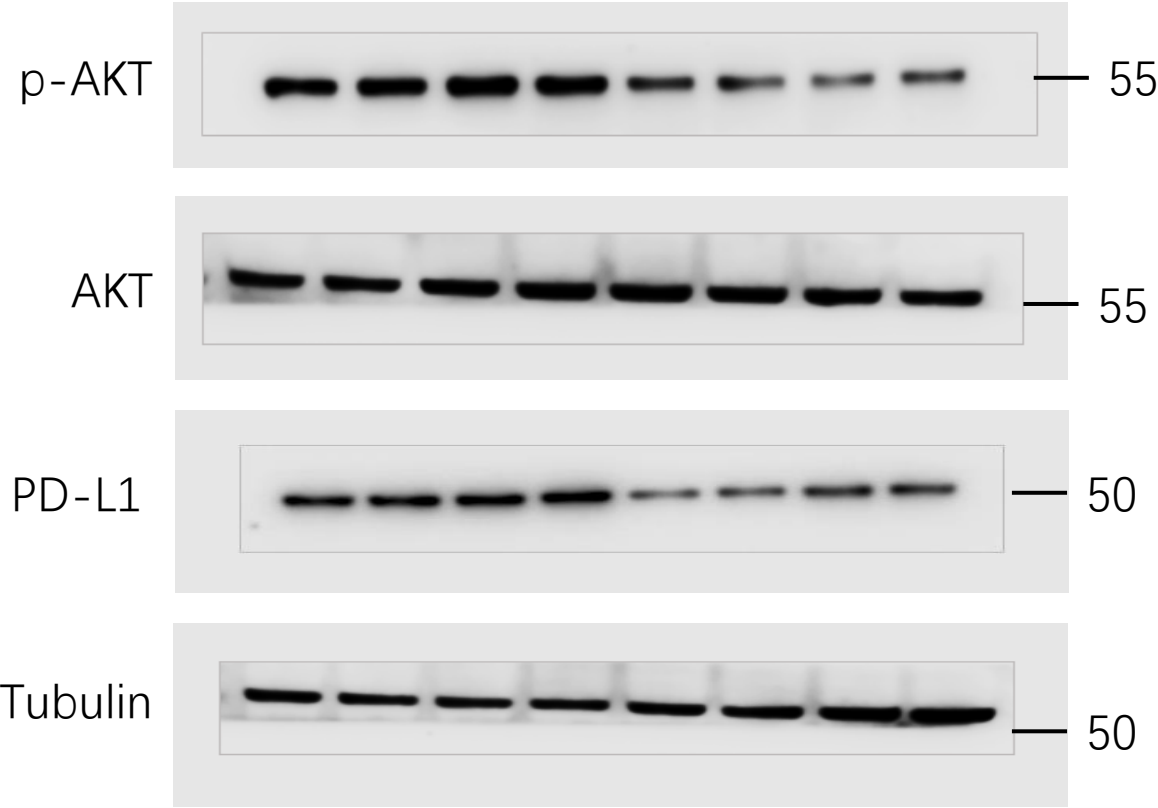

Figure 5M

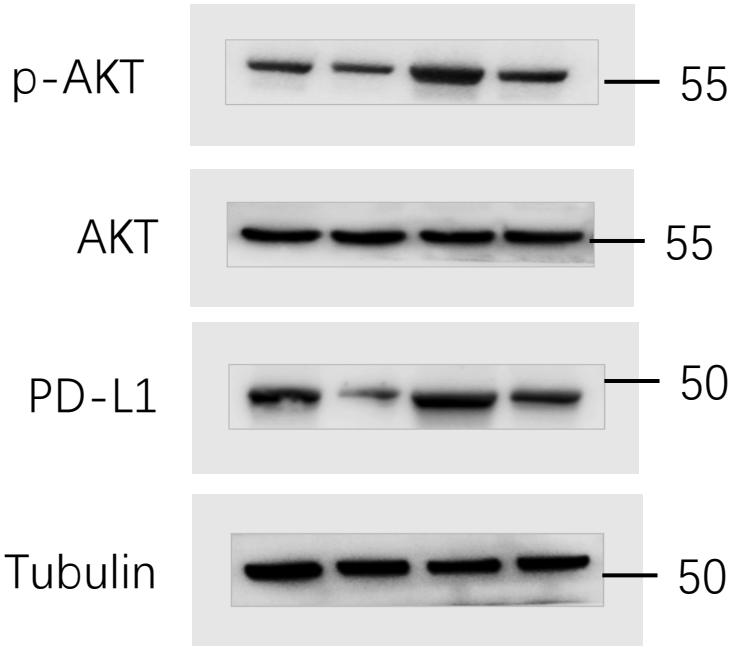

Figure 6B

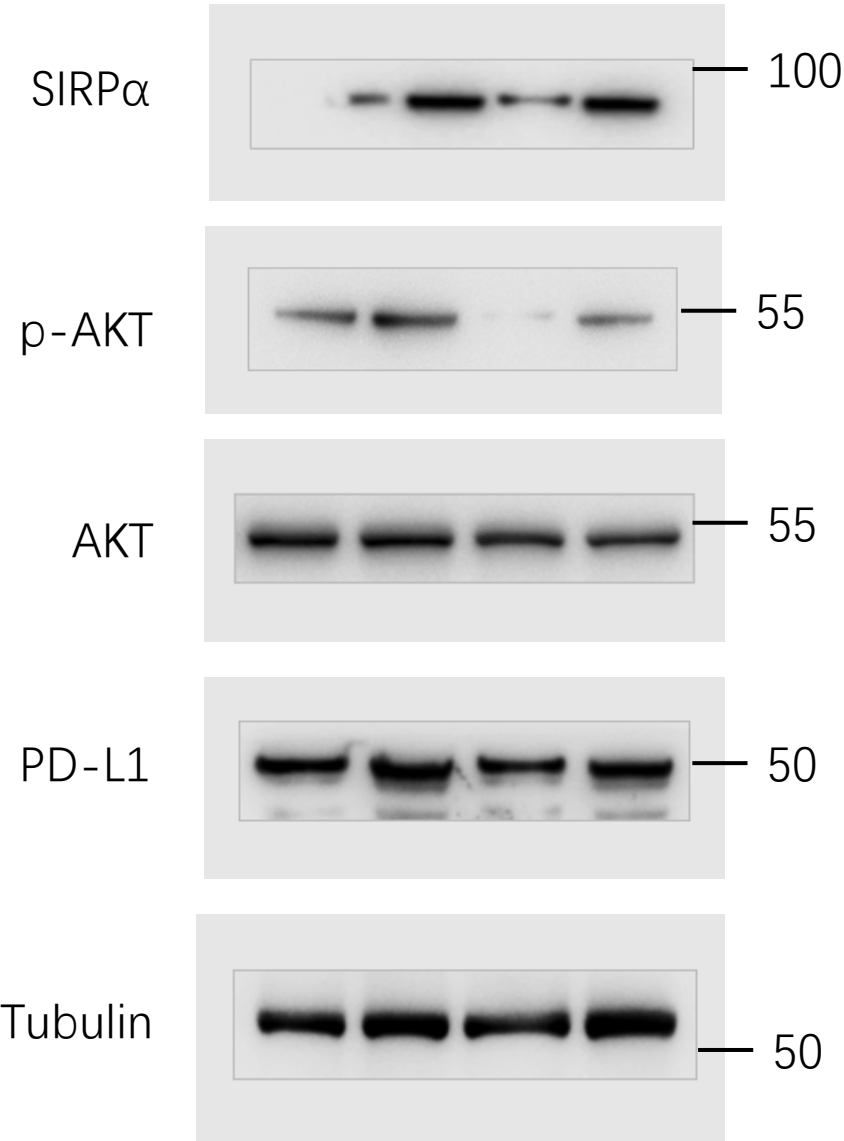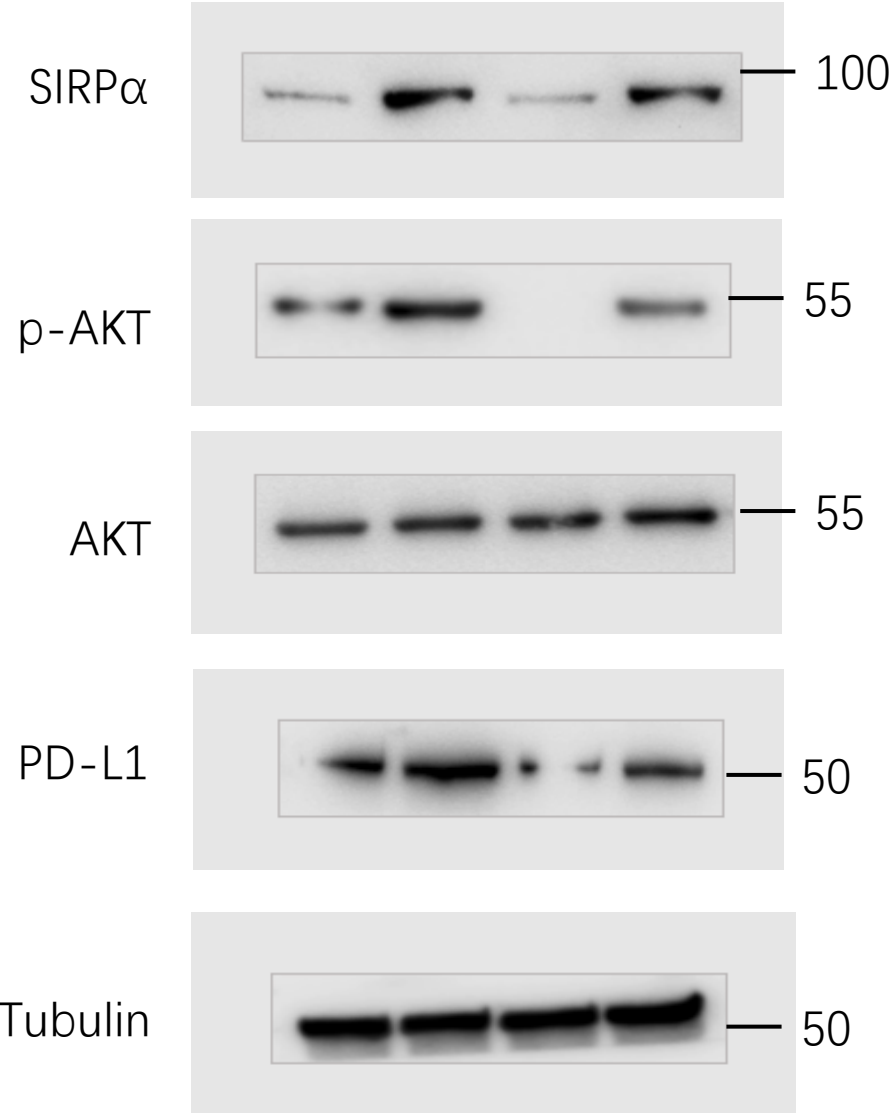

Supplement: Supplementary file 4 — original data [file 41419_2025_7779_MOESM4_ESM.pdf]
